# Design and Synthesis of New α-hydroxy β-fluoro/β-trifluoromethyl and Unsaturated Phosphonates from Carbohydrate-Derived Building Blocks via Pudovik and Horner–Wadsworth–Emmons Reactions †

**DOI:** 10.3390/molecules27175404

**Published:** 2022-08-24

**Authors:** Monika Bilska-Markowska, Wojciech Jankowski, Marcin Hoffmann, Marcin Kaźmierczak

**Affiliations:** 1 Faculty of Chemistry, Adam Mickiewicz University, Uniwersytetu Poznańskiego 8, 61-614 Poznań, Poland; 2 Centre for Advanced Technologies, Adam Mickiewicz University, Uniwersytetu Poznańskiego 10, 61-614 Poznań, Poland

**Keywords:** fluorinated *α*-hydroxyphosphonates, fluorinated unsaturated phosphonates, fluorinated building blocks, Pudovik, Horner–Wadsworth–Emmons

## Abstract

Herein, we present the application of fluorinated carbohydrate-derived building blocks for α-hydroxy β-fluoro/β-trifluoromethyl and unsaturated phosphonates synthesis. Pudovik and Horner–Wadsworth–Emmons reactions were applied to achieve this goal. The proposed pathway of the key reactions is supported by the experimental results, as well as quantum chemical calculations. The structure of the products was established by spectroscopic (1D, 2D NMR) and spectrometric (MS) techniques. Based on our data received, we claim that the progress of the Pudovik and HWE reactions is significantly influenced by the acidic protons present in the molecules as assessed by pK_a_ values of the reagent.

## 1. Introduction

**Figure 1:**
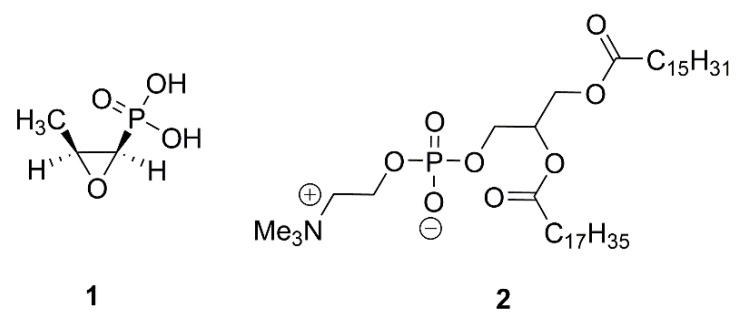
Structures of Fosfomycin **1** and Phosphatidylcholine **2**.

The synthesis of biologically significant compounds is based mainly on the ability to introduce an appropriate group of atoms into a target molecule [1,2]. An interesting approach relevant to this case may be introduction of fluorine containing groups into organic molecules. The relatively small size and electron properties of fluorine gave it a versatile use as a bioisostere and hence it is commonly applied as a substitute for a hydrogen atom. In addition, it is also an excellent mimetic of a carbonyl group, for example in peptide chains [3,4,5,6]. Nature has not endowed us with numerous fluorinated organic derivatives [7,8]. For this reason, synthesis in this area is the only way to obtain such molecules. Due to its remarkable chemical and physical properties, fluorine possessing compounds play an important role in biologically active molecules. This is particularly important in the aspect of obtaining new drugs [9,10,11,12,13,14,15]. Phosphonic acid derivatives are also among the known and used drugs (Fosfomycin **1**, Figure 1). These derivatives have found application as analogues of phosphates commonly occurring in living organisms and involved in important biological processes (component of cell membranes—Phosphatidylcholine **2**, Figure 1) [16,17]. Replacement of a bridging oxygen atom (R-O-P) in a phosphate group by a methylene group (-CH_2_-) can have significant biological effects. This modification may affect greater stability because carbon-phosphorus bond cannot be hydrolyzed by the enzyme that cleaves O-phosphate esters. For this reason, these isosteric analogues can be better substrates due to their similarity in size and shape to natural phosphates [18].

**Figure 2:**
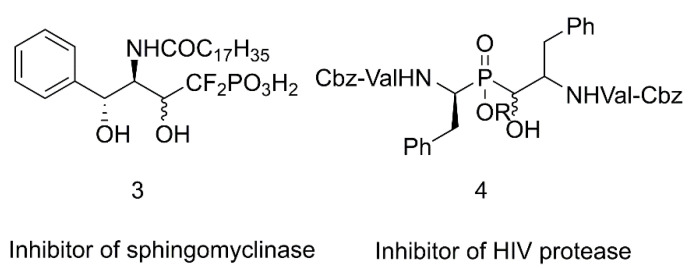
Examples of synthetic hydroxyphosphonates [19].

In the literature, there are many examples of various properties and biological activities of phosphonic acid derivatives containing a hydroxyl group [19,20] and/or fluorine atom in *α* position to a phosphonate group [21,22,23,24,25,26]. These compounds are also isosteric analogues of natural products [27] and enzyme inhibitors (Figure 2) [19,28]. There are also examples of phosphonate derivatives having a fluorine atom in a *β* position to a phosphorus atom [24,29,30] and fluorinated or nonfluorinated unsaturated phosphonates [31,32,33,34,35,36,37,38].

**Scheme 1:**
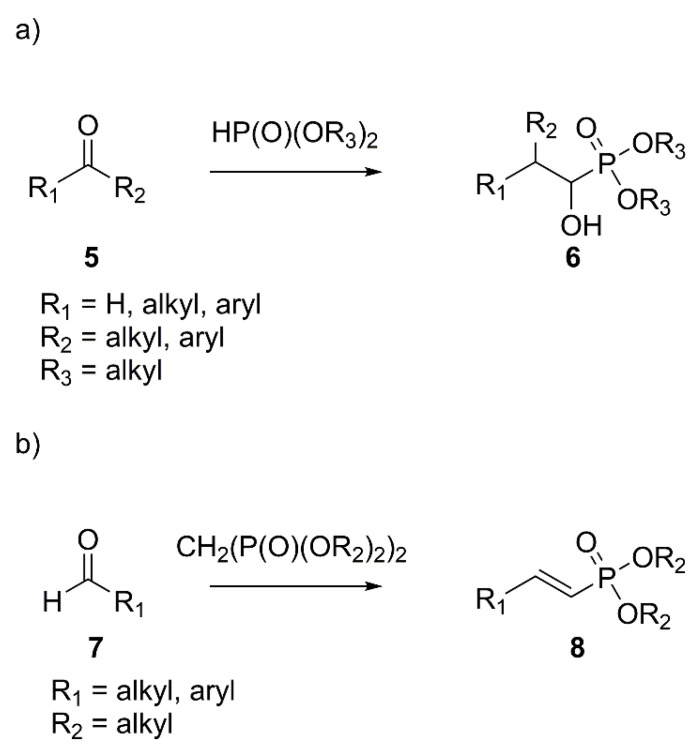
Synthetic routes to: (**a**) α-hydroxyphosphonates, (**b**) unsaturated phosphonates.

The most reliable approaches widely used in the synthesis of both fluorine-containing and non-fluorinated phosphonate derivatives are the Pudovik reaction (a) and the Horner–Wadsworth–Emmons olefination (b) (Scheme 1). The most frequently used method of *α*-hydroxyphosphonates synthesis is addition of dialkyl phosphite to a carbonyl compound, named after its discoverer—the Pudovik reaction [39,40]. HWE is a very highly stereoselective method for preparation of not only *α*,*β*-unsaturated phosphonates, but also other compounds, especially various types of natural product derivatives [41,42,43,44,45].

An efficient approach towards the synthesis of fluoro-containing compounds is through modular assembly using fluorinated building blocks. In the recent study we investigated application of the Claisen rearrangement reaction for construction of fluorine-containing scaffolds [46]. Carbohydrates were used as starting compounds, which are a very valuable source of building blocks, as well as chiral auxiliaries [47,48,49,50,51,52]. Specific intent of our present research effort is using early obtained carbohydrate-derived building blocks to receive new α-hydroxy β-fluoro/β-trifluoromethyl and unsaturated phosphonates via Pudovik and Horner–Wadsworth–Emmons reactions. The modifiable functional groups of our newly synthesized compounds give access for their further transformations. They can be considered feasible tools for more complex molecule design.

## 2. Results and Discussion

**Scheme 2:**
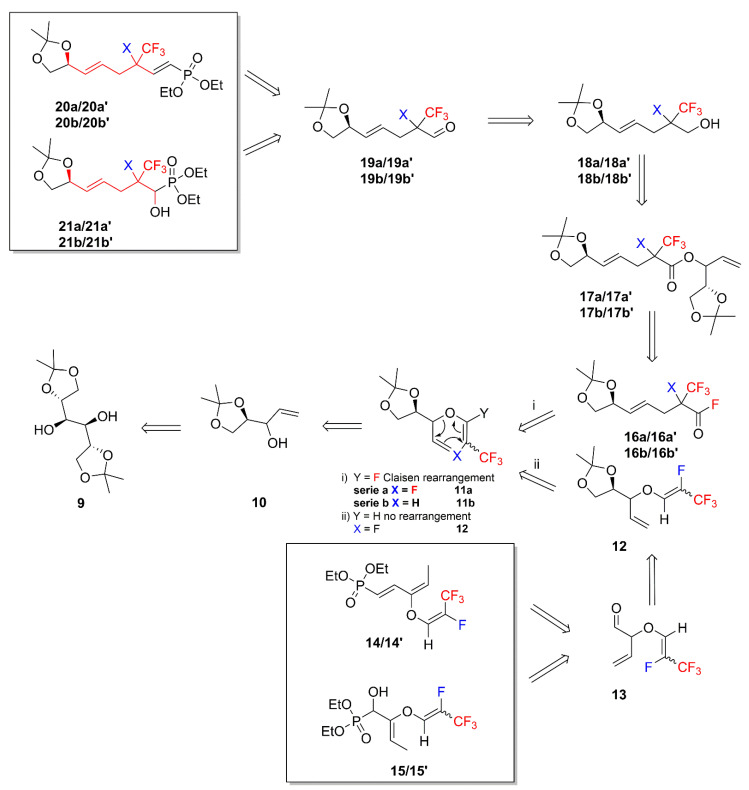
Synthetic strategy towards **14–15**, **20–21**.

In the present studies we report synthesis of fluorine containing phosphonates, **14–15**, **20–21**. 1,2;5,6-Di-*O*-isopropylidene-*α*-d-mannitol **9** was used as a starting material and successfully converted into **14–15**, **20–21** via **12** and **18** (Scheme 2). Fluorinated ethers **11** and **12** were prepared using a strategy previously developed in our research group [46,48].

**Scheme 3:**
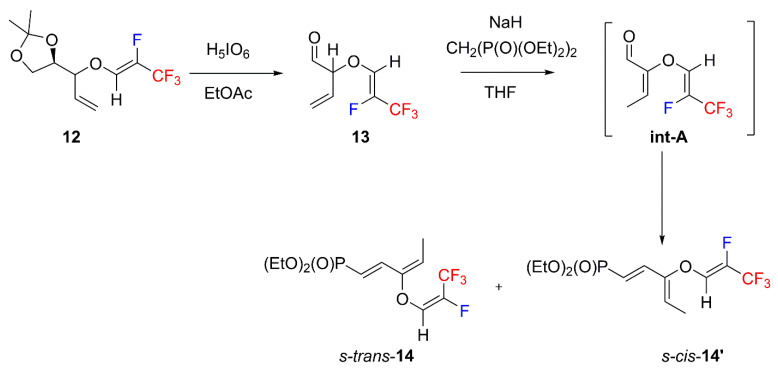
Synthesis of **14**.

Allyl-vinyl ethers **12**, as well as scaffolds bearing fluorine atoms resulting from Claisen rearrangement **18** were used as excellent precursors for the synthesis of fluorinated phosphonates (Scheme 2). The former **12**, depending on the olefin employed, can be yielded as a single *Z* isomer or a *Z*/*E* mixture, and readily transformed to a carbonyl compound **13** by reacting with periodic acid. The aldehyde **13** after evaporation of solvent was pure enough be subjected to subsequent reactions. For this purpose, tetraethyl methylenebisphosphonate was converted into appropriate anion with NaH and proceeded with pure *Z* isomer of **13** to HWE protocol (Scheme 3). The reaction was carried out with 1.75 eq of both tetraethyl methylenebisphosphonate and NaH in THF, for 15 h at room temperature. Surprisingly, the reaction did not progress according to our assumption. On the basis of ^1^H, ^13^C NMR spectra of the obtained product **14**, it can be concluded that in the initial stage of the process, under basic conditions, the substrate **13** isomerized to intermediate compound **int-A**, and afterwards the expected HWE reaction took place. In the following Scheme 3, a pathway of the discussed transformation is proposed. The observed 1,3-shift in the first step of the overall process is related to the presence of an acidic proton in the *α* position to the carbonyl group of fluorinated *β*,*γ*-unsaturated aldehyde **13**, which can be abstracted under reaction conditions. In the literature, a few examples of this type of isomerization of carbonyl compounds can be found, some of them occurring even with very weak bases. [53,54,55] However, to the best of our knowledge, this is the first known example of such a 1,3-shift involving *β*,*γ*-unsaturated aldehydes. As a result of HWE reaction, between *α*,*β*-unsaturated aldehyde **int-A** and nucleophilic derivative of tetraethyl methylenebisphosphonate, an *α*,*β*-unsaturated phosphonate with a fluorinated allyl group **14** was synthesized. In spectra of purified product **14**, two sets of signals in the ratio 1:0.2 were observed. It is caused by the presence of two diene conformers: “*s-cis*” **14′** and “*s-trans*” **14**. Geometry of the double bond located directly to the phosphorus atom was determined based on the NMR analysis, and is consistent with the expectations of the HWE outcome where the *E* isomer should be formed as the main product. [56] A coupling constant value was crucial for the analysis- it was found to be 17 Hz, which confirmed the *trans* relationship of the protons across the double bond.

**Scheme 4:**
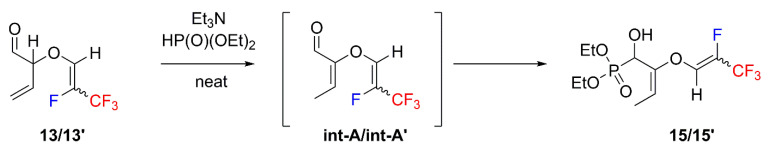
Synthesis of **15**.

**Figure 3:**
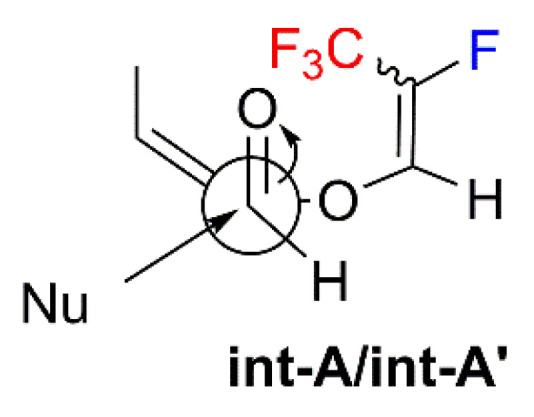
Felkin-Ahn model.

In order to study the reactivity and diastereoselectivity of the reaction with another nucleophilic agent, this time a mixture of *Z*/*E* isomers of aldehyde **13** was subjected to the Pudovik reaction (Scheme 4). This time the reaction was carried out under very mild conditions, without a solvent, and in the presence of stoichiometric amounts of diethyl phosphite, and triethylamine at room temperature. The reaction, as described previously, proceeded in an alkaline environment. In the first step, the double bond rearrangement was observed, and then followed by the actual nucleophilic attack. The Pudovik reaction was highly stereoselective, such a great selectivity can be explained by the Felkin-Ahn model presented in Figure 3.

Having results of both reactions, it can be concluded that the progress of those transformations is strongly dependent on the presence of an acidic proton in the α position of the carbonyl group in **13**.

In the present work we show a second class of molecules that we functionalized- these are the previously obtained by Claisen rearrangement *α*-fluoro-*α*-trifluoromethyl-*γ*,*δ*-unsaturated **18a/18a′** (1:0.65 ratio) and *α*-trifluoromethyl-*γ*,*δ*-unsaturated **18b/18b′** (1:1 ratio) alcohols [46]. These compounds were oxidized with quantitative yields to the corresponding aldehydes **19a/19a′** and **19b/19b′** using Dess-Martin reagent. The reactions were carried out under mild conditions at room temperature. Complete disappearance of the substrates and selective formation of new products was observed after only 5 min. Aldehydes **19a/19a′** and **19b/19b′** were isolated by simple extraction, and were pure enough to be subjected to further transformations. Both fluorinated carbonyl compounds **19a/19a′**, **19b/19b′** were subjected to HWE and Pudovik reactions under the same conditions as described above as well.

**Scheme 5:**
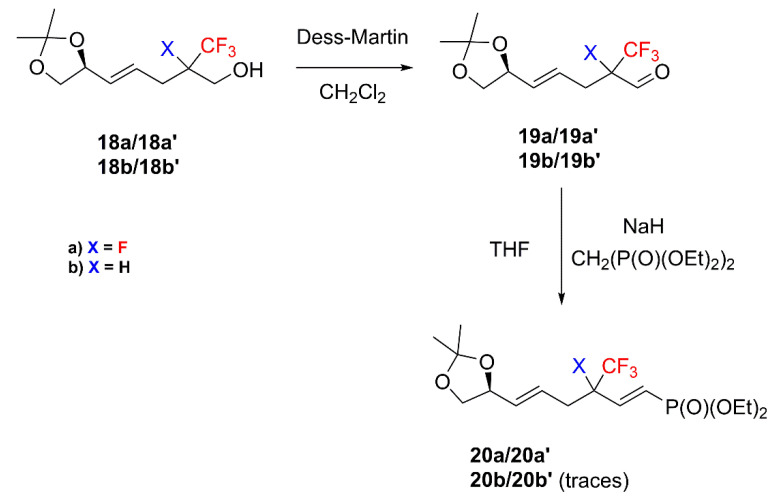
Synthesis of **20**.

The HWE reaction carried out on *α*-fluoro-*α*-trifluoromethyl-*γ*,*δ*-unsaturated **19a/19a′** derivatives proceeded as expected and yielded *E* olefins **20a/20a′** as the main products (Scheme 5). In this case the coupling constant between the protons located at the newly produced double bond was also 17 Hz. Unfortunately, an analogous reaction with *α*-trifluoromethyl-*γ*,*δ*-unsaturated **19b/19b′** aldehydes provided only traces of the expected HWE products **20b/20b′**. These compounds were characterized on the basis of ^1^H, ^19^F, ^31^P and HRMS spectra. **20b/20b′** occurred to be unstable during prolonged NMR experiments (^13^C, 2D NMR). Perhaps, the initial abstraction of acidic proton occurs, and then highly electrophilic and reactive structures are formed.

**Scheme 6:**
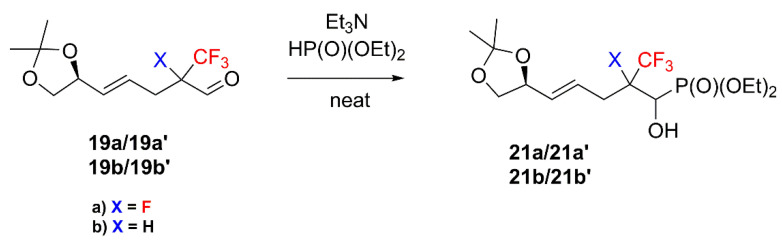
Synthesis of **21**.

Aldehydes **19a/19a′** and **19b/19b′** were also subjected to the Pudovik reaction (Scheme 6). When the derivatives containing an *α*-fluoro-*α*-trifluoromethyl-*γ*,*δ*-unsaturated moiety **19a/19a′** were used as substrates, the corresponding hydroxy phosphonates **21a/21a′** were obtained with good yield. A very high diastereoselectivity of the process was observed this time. The reaction led to only one new stereogenic center on a carbon atom bonded to a phosphorus atom. The reaction with the second aldehydes **19b/19b′**, resulted in a slight reduction in the yield of the products **21b/21b′**. This may also be related to the presence of an acidic proton located adjacent to the -CF_3_ group.

In order to explain and investigate the unexpected reactions of aldehydes **13** and **19b/19b′**, we decided to perform quantum mechanical calculations to determine their pK_a_ values.

DFT Calculations

**Figure 4:**
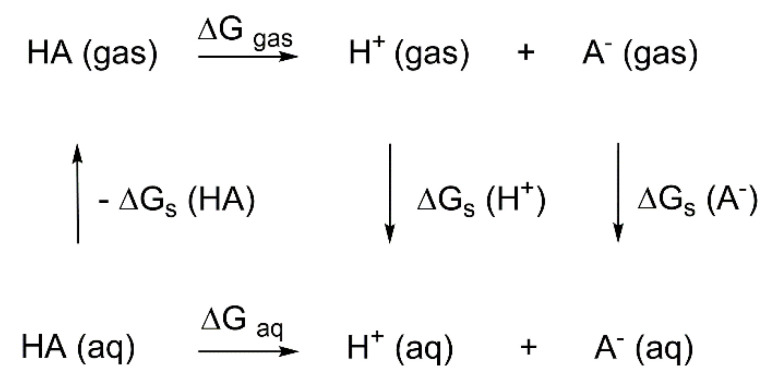
Thermodynamic cycle used for pK_a_ calculation.

The aim of the calculations was to obtain calculated value of pK_a_ to estimate the acid strength of 2-((2,3,3,3-tetrafluoroprop-1-en-1-yl)oxy)but-3-enal **13** and α-trifluoromethyl-*γ*,*δ*-unsaturated aldehyde **19b** in comparison to other organic acids whose pK_a_ were measured in aqueous solution. Energy calculations were performed within DFT framework at M062X/6-31+G(d) level of theory [57,58]. This method and basis set was used by Zeng et al. [59] for similar calculations which resulted in calculated values of pK_a_. We also conducted frequency analysis [60] to access thermochemistry data needed, and to verify that the geometry optimization led to the potential energy minimum. Calculations were carried out in vacuo and aqueous solution with use of the Polarizable Continuum Model (PCM) [61]. All quantum mechanics calculations were performed with the GAUSSIAN 09 [62] available within Pl-Grid infrastructure. To calculate pK_a_ we used thermodynamic cycle depicted in Figure 4 below.

Such a type of thermodynamic cycle was recommended in papers by Liptak et al. and by Zeng et al. [59,63]. We applied methodology presented in those articles in our work. To calculate pK_a_ we used equation (Equation (1)) proposed by Zeng et al. [59] included below.

(1) $${\mathrm{pK}}_{a}=A\frac{\Delta G_{\mathrm{aq}}}{\mathrm{RTln}10}+B$$

where:

$\Delta G_{\mathrm{aq}}—\mathrm{solution}\;\mathrm{phase}\;\mathrm{free}\;\mathrm{energy}$

R—gas constant

T—temperature

A & B—linear parameters

Parameters A and B were determined on the basis of known pK_a_ values for five chemical compounds: formic acid, trifluoroacetic acid, dimethyl malonate, acetone and chloroacetone. These compounds were also optimized in vacuo and water solution, and on the basis of information gathered from frequency analyses we were able to calculate pK_a_ values for each structure. Calculated and known experimental values of pK_a_ of formic acid, trifluoroacetic acid, dimethyl malonate, acetone and chloroacetone are gathered in Table 1 below.

As shown in Table 1 values of calculated pK_a_ differed slightly from experimentally measured values of pK_a_ (values of empirical parameters A and B needed in Equation (1). were 0.3541 and −0.3387 for parameters A and B, respectively). The calculated values of pK_a_ for 2-((2,3,3,3-tetrafluoroprop-1-en-1-yl)oxy)but-3-enal **13** and *α*-trifluoromethyl-*γ*,*δ*-unsaturated aldehyde **19b** were 13.3 and 15.1, respectively. Obtained pK_a_ values suggest that acidity of **13** is similar to acidity of dimethyl malonate (experimental pK_a_ value 13.5), while acidity of **19b** is greater than the acidity of chloroacetone (experimental pK_a_ value 16.5). Examples of use of, weak bases such as K_2_CO_3_, Et_3_N, and NaH to abstract acidic protons from dimethyl malonate or chloroacetone can be found in the literature. [65,66,67,68] Thus, the obtained calculated results of pK_a_ values of **13** and **19b** correlate with experimental observations, in which under the reaction conditions the abstraction of acidic proton can be observed in the first step of the reaction.

## 3. Conclusions

In conclusion, starting from conveniently obtained via Claisen rearrangement fluorinated scaffolds **18** or allyl-vinyl ethers **12**, new *α*-hydroxy *β*-fluoro/*β*-trifluoromethyl and unsaturated phosphonates have been achieved. The key reactions proceeded under mild conditions with good yields. The unexpected progress of the reactions has been correlated with quantum mechanical calculations and indicates connection with pK_a_ values of the molecules **13** and **19b/19b′**. To the best of our knowledge of the ever-growing interest in fluorinated phosphonates as enzyme inhibitors, the compounds obtained in this research can serve as material for future synthetic studies. These compounds have been also prepared as examples to illustrate the synthetic potential of fluorinated derivatives **12** and **18**.

## 4. Materials and Methods

### 4.1. General Methods

^1^H NMR, ^13^C NMR, ^19^F NMR, ^31^P NMR and 2D-NMR spectra were performed on Bruker ASCEND 400 (400 MHz) and Bruker ASCEND 600 (600 MHz) spectrometers. Chemical shifts of ^1^H NMR were expressed in parts per million downfield from TMS as an internal standard (δ = 0) in CDCl_3_. Chemical shifts of ^13^C NMR were expressed in parts per million downfield and upfield from CDCl_3_ as an internal standard (δ = 77.0). Chemical shifts of ^19^F NMR were expressed in parts per million upfield from CFCl_3_ as an internal standard (δ = 0) in CDCl_3_. ^31^P NMR chemical shifts were expressed in parts per million in CDCl_3_. High-resolution mass spectra were recorded by electron spray (MS-ESI) technique using QToF Impact HD Bruker spectrometer. Reagent grade chemicals were used and solvents were dried by refluxing with sodium metal-benzophenone (THF) and distilled under an argon atmosphere. All moisture sensitive reactions were carried out under an argon atmosphere using oven-dried glassware. Reaction temperatures below 0 °C were obtained using a bath cooling (dry ice/iso-propanol). Thin-layer chromatography (TLC) was performed on Merck Kieselgel 60-F254 with EtOAc/hexane as developing systems. Visualization of the reactions products was achieved using UV light (254 nm) and a standard procedure (solution of phosphomolybdenic acid or solution of potassium permanganate). Merck Kieselgel 60 (230–400 mesh) was used for column chromatography (more detailed data in Supplementary Materials).

### 4.2. General Procedure of Periodic Acid Oxidation Synthesis of ***13***

A solution of **12**/**12′** (1 equiv.) and H_5_IO_6_ (2 equiv.) in EtOAc (5 mL) was stirred for 4.5 h. The mixture was filtered, and the filtrate was evaporated to give the crude product as a creamy oil. The crude product was used for the next step [69].

### 4.3. General procedure of Dess-Martin oxidation Synthesis of ***19***

The Dess-Martin periodinane (1.2 equiv.) was added to a stirred solution of **18** (1 equiv.) in 5 mL of CH_2_Cl_2_ at RT. After 5 min the solution was diluted with CH_2_Cl_2_ and a mixture of sat. NaHCO_3_ and aq. Na_2_S_2_O_3_ (1:1) 5 mL. After stirring for 5 min the phases were separated. Organic phases were dried (Na_2_SO_4_) and concentrated to give oils which were used without further purification [70].

### 4.4. General Procedure of HWE Reaction Synthesis of ***14*** and ***20***

Tetraethyl methylenediphosphonate (1.75 equiv.) was added dropwise to a mixture of sodium hydride (60% dispersion in oil, 1.75 equiv.) in 2 mL of dry THF at 0 °C under argon. The reaction mixture was stirred for 10 min at 0 °C and then stirred at room temperature for 20 min. The reaction mixture was cooled to 0 °C, a solution of aldehyde **13**/**13′** (1.75 equiv.) in 1 mL of dry THF was added, and the resulting solution was stirred at room temperature for 15 h. The reaction mixture was concentrated, and the residue was purified using column chromatography (*n*-hexane/ethyl acetate 90:10 → 50:40) [71].

Diethyl ((1*E*,3*E*)-3-(((*E*)-2,3,3,3-tetrafluoroprop-1-en-1-yl)oxy)penta-1,3-dien-1-yl)phosphonate **(14/14′):** Colorless oil (73 mg, 39 %, ratio 1:0.2): Major: ^1^H NMR (400 MHz, Chloroform-*d*) *δ* = 6.95 (dd, *J* = 21.9, 17.0 Hz, 1H, PC*H*=CH), 6.39 (dq, *J* = 18.5, 1.1 Hz, 1H, OC*H*=CFCF_3_), 5.81 (t, *J* = 17.3 Hz, 1H, PCH=C*H*), 5.66 (dq, *J* = 7.2, 1.7 Hz, 1H, =C*H*CH_3_), 4.13 (q, *J* = 7.1 Hz, 2H, OC*H*_2_CH_3_), 1.83 (d, *J* = 7.2 Hz, 3H, =CHC*H*_3_), 1.34 (t, *J* = 7.0 Hz, 3H, OCH_2_C*H*_3_). ^13^C NMR (101 MHz, Chloroform-*d*) *δ* = 151.33 (d, *J* = 25.0 Hz, CH*=C*O), 142.00 (d, *J* = 7.8 Hz, O*C*H=CFCF_3_), 134.34 (dq, *J* = 251.9, 39.4 Hz, OCH=*C*FCF_3_), 132.89–132.66 (m, PCH=*C*H), 123.54 (s, *C*H=CH_3_), 119.52 (dq, *J* = 269.1, 34.5 Hz, *C*F_3_), 114.10 (d, *J* = 191.8 Hz, P*C*H=CH), 62.18 (d, *J* = 5.6 Hz, O*C*H_2_CH_3_), 61.98 (d, *J* = 5.5 Hz, O*C*H_2_CH_3_), 16.28 (s, 2 × OCH_2_*C*H_3_). 11.52 (s, *C*H_3_). ^31^P NMR (162 MHz, Chloroform-*d*) *δ* = 18.33 (s). ^19^F NMR (376 MHz, Chloroform-*d*) *δ* = −71.47 (d, *J* = 14.7 Hz, C*F*_3_), −160.73 (dq, *J* = 18.2, 14.5 Hz, *F*). Minor: ^1^H NMR (400 MHz, Chloroform-*d*) *δ* = 6.92 (dd, *J* = 21.8, 17.1 Hz, 1H, PC*H*=CH), 6.53 (dq, *J* = 18.1, 1.1 Hz, 1H, OC*H*=CFCF_3_), 5.99 (t, *J* = 16.9 Hz, 1H, PCH=C*H*), 5.67 (dq, *J* = 7.2, 2.0 Hz, 1H, =C*H*CH_3_), 4.10 (q, *J* = 7.2 Hz, 2H, OC*H*_2_CH_3_), 2.07 (d, *J* = 8.3 Hz, 3H, =CHC*H*_3_), 1.26 (t, *J* = 7.1 Hz, 3H, OCH_2_C*H*_3_). ^13^C NMR (101 MHz, Chloroform-*d*) *δ* = 152.04 (d, *J* = 25.0 Hz, CH*=C*O), 140.65 (d, *J* = 7.8 Hz, O*C*H=CFCF_3_), 134.34 (dq, *J* = 251.9, 39.4 Hz, OCH=*C*FCF_3_), 132.39–132.24 (m, PCH=*C*H), 120.56 (s, *C*H=CH_3_), 119.52 (dq, *J* = 269.1, 34.5 Hz, *C*F_3_), 118.23 (d, *J* = 190.6 Hz, P*C*H=CH), 62.18 (d, *J* = 5.6 Hz, O*C*H_2_CH_3_), 61.98 (d, *J* = 5.5 Hz, O*C*H_2_CH_3_), 16.22 (s, 2 × OCH_2_*C*H_3_). 11.50 (s, *C*H_3_). ^31^P NMR (162 MHz, Chloroform-*d*) *δ* = 16.82 (s). ^19^F NMR (376 MHz, Chloroform-*d*) *δ* = −71.59 (d, *J* = 14.6 Hz, C*F*_3_), −159.14 (dq, *J* = 18.4, 14.6 Hz, *F*). HRMS (ESI) calcd. for C_12_H_18_F_4_O_4_P ([M + H]^+^): 333.0879, found: 333.0873.

Diethyl ((1*E*,5*E*)-6-((*S*)-2,2-dimethyl-1,3-dioxolan-4-yl)-3-fluoro-3-(trifluoromethyl)hexa-1,5-dien-1-yl)phosphonate (**20a/20a′**)**:** Colorless oil (82 mg, 72%, ratio:1:0.65): Major: ^1^H NMR (400 MHz, Chloroform-*d*) *δ* = 6.62 (dddd, *J* = 23.0, 20.4, 17.1, 6.2 Hz, 1H, C*H*=CHP), 6.19 (t, *J* = 17.1, 1H, CH=C*H*P), 5.64 (dd, *J* = 15.6, 6.2 Hz, 1H, CH=C*H*CH_2_), 5.61 (dd, *J* = 15.5, 6.0 Hz, 1H, C*H*=CHCH_2_), 4.46 (q, *J* = 7.6 Hz, 1H, C*H*CH=CH), 4.16–4.03 (m, 4H, 2 × OC*H*_2_CH_3_), 4.06 (dd, *J* = 8.2, 6.1 Hz, 1H, OC*H*H), 3.56 (t, *J* = 8.1 Hz, 1H, OCH*H*), 2.89–2.74 (m, 1H, C*H*HCF), 2.71–2.51 (m, 1H, CH*H*CF), 1.40 (s, 3H, C*H*_3_), 1.36 (s, 3H, C*H*_3_), 1.35–1.32 (m, 6H, 2 × OCH_2_C*H*_3_). ^13^C NMR (101 MHz, Chloroform-*d*) *δ* = 141.44 (dd, *J* = 10.6, 7.8 Hz, *C*H=CHP), 134.77 (s, *C*H=CHCH_2_), 123.40 (dd, *J* = 185.7, 8.8 Hz, CH=*C*HP), 123.08 (d, *J* = 4.2 Hz, CH=*C*HCH_2_), 122.36 (dq, *J* = 284.3, 30.3 Hz, *C*F_3_), 109.45 (s, *C*(CH_3_)_2_), 93.76 (dq, *J* = 194.4, 31.2 Hz, *C*F), 76.24 (s, *C*HCH=CH), 69.27 (s, O*C*H_2_), 62.34 (d, *J* = 5.5 Hz, 2 × O*C*H_2_CH_3_), 35.16 (d, *J* = 22.3 Hz, *C*H_2_CF), 26.58 (s, *C*H_3_), 25.81 (s, *C*H_3_), 16.40–16.22 (m, 2 × OCH_2_*C*H_3_). ^31^P NMR (162 MHz, Chloroform-*d*) *δ* = 14.96 (s). ^19^F NMR (376 MHz, Chloroform-*d*) *δ* = −80.24 (d, *J* = 7.3 Hz, C*F*_3_), −177.00 (dq, *J* = 7.1, 2.2 Hz, *F*). Minor: ^1^H NMR (400 MHz, Chloroform-*d*) *δ* = 6.62 (dddd, *J* = 23.0, 20.4, 17.1, 6.2 Hz, 1H, C*H*=CHP), 6.19 (t, *J* = 17.1, 1H, CH=C*H*P), 5.64 (dd, *J* = 15.6, 6.2 Hz, 1H, CH=C*H*CH_2_), 5.61 (dd, *J* = 15.5, 6.0 Hz, 1H, C*H*=CHCH_2_), 4.48 (q, *J* = 7.9 Hz, 1H, C*H*CH=CH), 4.16–4.03 (m, 4H, 2 × OC*H*_2_CH_3_), 4.06 (dd, *J* = 8.2, 6.1 Hz, 1H, OC*H*H), 3.56 (t, *J* = 8.1 Hz, 1H, OCH*H*), 2.89–2.74 (m, 1H, C*H*HCF), 2.71–2.51 (m, 1H, CH*H*CF), 1.41 (s, 3H, C*H*_3_), 1.38 (s, 3H, C*H*_3_), 1.35–1.32 (m, 6H, 2 × OCH_2_C*H*_3_). ^13^C NMR (101 MHz, Chloroform-*d*) *δ* = 141.29 (dd, *J* = 11.0, 8.2 Hz, *C*H=CHP), 134.83 (s, *C*H=CHCH_2_), 128.48 (dd, *J* = 186.8, 9.0 Hz, CH=*C*HP), 122.96 (d, *J* = 4.0 Hz, CH=*C*HCH_2_), 122.36 (dq, *J* = 284.3, 30.3 Hz, *C*F_3_), 109.51 (s, *C*(CH_3_)_2_), 93.63 (dq, *J* = 194.4, 31.3 Hz, *C*F), 76.22 (s, *C*HCH=CH), 69.27 (s, O*C*H_2_), 62.29 (d, *J* = 5.7 Hz, O*C*H_2_CH_3_), 62.20 (d, *J* = 5.7 Hz, O*C*H_2_CH_3_), 35.15 (d, *J* = 19.8 Hz, *C*H_2_CF), 26.58 (s, *C*H_3_), 25.80 (s, *C*H_3_), 16.40–16.22 (m, 2 × OCH_2_*C*H_3_). ^31^P NMR (162 MHz, Chloroform-*d*) *δ* = 14.95 (s). ^19^F NMR (376 MHz, Chloroform-*d*) *δ* = −80.27 (d, *J* = 7.3 Hz, C*F*_3_), −176.85 (dq, *J* = 7.0, 2.1 Hz, *F*). HRMS (ESI) calcd. for C_16_H_25_F_4_O_5_PNa ([M + Na]^+^): 427.1273, found: 427.1270.

Diethyl ((1*E*,5*E*)-6-((*S*)-2,2-dimethyl-1,3-dioxolan-4-yl)-3-(trifluoromethyl)hexa-1,5-dien-1-yl)phosphonate (**20b/20b′**): Colorless oil (8 mg, 16 %, ratio 1:0.9): Major: ^1^H NMR (400 MHz, Chloroform-*d*) *δ* = 6.87 (dd, *J* = 29.4, 1.9 Hz, 1H, C*H*=CHP), 5.72 (t, *J* = 17.2, 1H, CH=C*H*P), 5.80–5.66 (m, 1H, CH=C*H*CH_2_), 5.48 (dd, *J* = 15.4, 7.4 Hz, 1H, C*H*=CHCH_2_), 4.48 (q, *J* = 7.0 Hz, 1H, C*H*CH=CH), 4.09 (q, *J* = 6.9 Hz, 4H, 2 × OC*H*_2_CH_3_), 4.08 (dd, *J* = 8.1, 6.2 Hz, 1H, OC*H*H), 3.55 (q, *J* = 8.2 Hz, 1H, C*H*CF_3_), 3.52 (t, *J* = 7.9 Hz, 1H, OCH*H*), 3.20–2.94 (m, 2H, C*H_2_*CHCF_3_), 1.41 (s, 3H, C*H*_3_), 1.38 (s, 3H, C*H*_3_), 1.33 (t, *J* = 7.0 Hz, 6H, 2 × OCH_2_C*H*_3_). ^31^P NMR (162 MHz, Chloroform-*d*) *δ* = 18.76–18.35 (m). ^19^F NMR (376 MHz, Chloroform-*d*) *δ* = −65.53 (s, 3F, C*F*_3_). Minor: ^1^H NMR (400 MHz, Chloroform-*d*) *δ* = 7.24 (dd, *J* = 22.3, 17.4 Hz, 1H, C*H*=CHP), 5.72 (t, *J* = 17.2, 1H, CH=C*H*P), 5.80–5.66 (m, 1H, CH=C*H*CH_2_), 5.62 (dd, *J* = 15.4, 7.4 Hz, 1H, C*H*=CHCH_2_), 4.45 (q, *J* = 7.1 Hz, 1H, C*H*CH=CH), 4.09 (q, *J* = 6.9 Hz, 4H, 2 × OC*H*_2_CH_3_), 4.07 (dd, *J* = 8.2, 7.2 Hz, 1H, OC*H*H), 3.55 (q, *J* = 8.2 Hz, 1H, C*H*CF_3_), 3.52 (t, *J* = 7.9 Hz, 1H, OCH*H*), 3.20–2.94 (m, 2H, C*H_2_*CHCF_3_), 1.40 (s, 3H, C*H*_3_), 1.37 (s, 3H, C*H*_3_), 1.33 (t, *J* = 7.0 Hz, 6H, 2 × OCH_2_C*H*_3_). ^31^P NMR (162 MHz, Chloroform-*d*) *δ* = 18.76–18.35 (m). ^19^F NMR (376 MHz, Chloroform-*d*) *δ* = −65.44 (s, 3F, C*F*_3_). HRMS (ESI) calcd. for C_16_H_26_F_3_O_5_PNa ([M + Na]^+^): 409.1368, found: 409.1371.

### 4.5. General Procedure of Pudovik Reaction Synthesis of ***15*** and ***21***

TEA (1.0 equiv.) was added under an argon atmosphere to a mixture of stirred solution of diethyl phosphite (1.0 equiv.) and an aldehyde **13** or **19** (1.0 equiv.). The reaction mixture was stirred at room temperature overnight, then was diluted with 20 mL of water and extracted with ethyl acetate (3 × 15 mL). The organic layers were washed with NaCl_sat._, dried over MgSO_4_ or Na_2_SO_4_, filtrated and concentrated under reduced pressure. The residue was purified using column chromatography (chloroform/methanol 100:0 → 100:0.5) [26].

Diethyl ((2*Z*)-1-hydroxy-2-((2,3,3,3-tetrafluoroprop-1-en-1-yl)oxy)but-2-en-1-yl)phosphonate **(15/15′)**: Colorless oil (46 mg, 49%, ratio 1:0.1): Major: ^1^H NMR (400 MHz, Chloroform-*d*) *δ* = 6.63 (dd, *J* = 19.0, 1.2 Hz, 1H, OC*H*=CFCF_3_), 5.50 (dq, *J* = 7.0, 3.6 Hz, 1H, =C*H*C*H*_3_), 4.75 (bs, O*H*), 4.46 (d, *J* = 12.5 Hz, 1H, PC*H*OH), 4.20 (q, *J* = 7.2 Hz, 2H, OC*H*_2_CH_3_), 1.72 (d, *J* = 7.1 Hz, 3H, =CHC*H*_3_), 1.34 (t, *J* = 7.1 Hz, 3H, OCH_2_C*H*_3_). ^13^C NMR (101 MHz, Chloroform-*d*) *δ* = 150.02 (d, *J* = 4.0 Hz, CH(OH)*=C*O), 134.00 (m, O*C*H=CFCF_3_), 133.53 (dq, *J* = 249.4, 39.2 Hz, OCH=*C*FCF_3_), 119.87 (dq, *J* = 268.8, 34.6 Hz, *C*F_3_), 114.09 (d, *J* = 9.4 Hz, *=C*HCH_3_), 68.98 (d, *J* = 161.5 Hz, *C*H(OH)*=*CO), 63.81 (d, *J* = 7.0 Hz, O*C*H_2_CH_3_), 63.23 (d, *J* = 7.3 Hz, O*C*H_2_CH_3_), 16.26 (d, *J* = 5.9 Hz, OCH_2_*C*H_3_), 16.29 (d, *J* = 5.9 Hz, OCH_2_*C*H_3_),10.43 (d, *J* = 1.5 Hz, *C*H_3_). ^31^P NMR (162 MHz, Chloroform-*d*) *δ* = 19.87 (s). ^19^F NMR (376 MHz, Chloroform-*d*) *δ* = −71.40 (d, *J* = 14.7 Hz, C*F*_3_), −163.22 (dq, *J* = 19.1, 15.0 Hz, *F*). Minor: ^1^H NMR (400 MHz, Chloroform-*d*) *δ* = 6.99 (d, *J* = 8.8 Hz, 1H, OC*H*=CFCF_3_), 5.50 (dq, *J* = 7.0, 3.6 Hz, 1H, =C*H*C*H*_3_), 4.75 (bs, O*H*), 4.46 (d, *J* = 12.5 Hz, 1H, PC*H*OH), 4.20 (q, *J* = 7.2 Hz, 2H, OC*H*_2_CH_3_), 1.71 (d, *J* = 6.9 Hz, 3H, =CHC*H*_3_), 1.35 (t, *J* = 6.6 Hz, 3H, OCH_2_C*H*_3_). ^13^C NMR (101 MHz, Chloroform-*d*) *δ* = 150.02 (d, *J* = 4.0 Hz, CH(OH)*=C*O), 134.00 (m, O*C*H=CFCF_3_), 133.53 (dq, *J* = 249.4, 39.2 Hz, OCH=*C*FCF_3_), 119.87 (dq, *J* = 268.8, 34.6 Hz, *C*F_3_), 114.09 (d, *J* = 9.4 Hz, *=C*HCH_3_), 68.91 (d, *J* = 161.4 Hz, *C*H(OH)*=*CO), 63.76 (d, *J* = 6.7 Hz, O*C*H_2_CH_3_), 63.19 (d, *J* = 7.4 Hz, O*C*H_2_CH_3_), 16.26 (d, *J* = 5.9 Hz, OCH_2_*C*H_3_), 16.29 (d, *J* = 5.9 Hz, OCH_2_*C*H_3_),10.35 (d, *J* = 1.6 Hz, *C*H_3_). ^31^P NMR (162 MHz, Chloroform-*d*) *δ* = 19.95 (s). ^19^F NMR (376 MHz, Chloroform-*d*) *δ* = −68.45 (d, *J* = 11.4 Hz, C*F*_3_), −180.98 (dq, *J* = 11.4, 8.9 Hz, *F*). HRMS (ESI) calcd. for C_11_H_17_F_4_O_5_PNa ([M + Na]^+^): 359.0647, found: 359.0648.

Diethyl ((*E*)-5-((*S*)-2,2-dimethyl-1,3-dioxolan-4-yl)-2-fluoro-1-hydroxy-2-(trifluoromethyl)pent-4-en-1-yl)phosphonate (**21a/21a′**): Colorless oil (68mg, 87 %, ratio 1:0.65): Major: ^1^H NMR (400 MHz, Chloroform-*d*) *δ* = 5.77–5.73 (m, 1H, CH=C*H*CH_2_), 5.61 (d, *J* = 6.8 Hz, 1H, C*H*=CHCH_2_), 4.67 (td, *J* = 7.3, 1H, O*H*), 4.44 (q, *J* = 7.1 Hz, 1H, C*H*CH=CH), 4.36–4.25 (m, 1H, C*H*P), 4.22–4.06 (m, 4H, 2 × OC*H*_2_CH_3_), 4.01 (dd, *J* = 8.2, 6.2 Hz, 1H, OC*H*H), 3.52 (dd, *J* = 7.9, 2.3 Hz, 1H, OCH*H*), 3.08–2.92 (m, 1H, C*H*HCF), 2.92–2.74 (m, 1H, CH*H*CF), 1.35 (s, 3H, C*H*_3_), 1.31 (s, 3H, C*H*_3_), 1.28 (t, *J* = 7.0 Hz, 6H, 2 × OCH_2_C*H*_3_). ^13^C NMR (101 MHz, Chloroform-*d*) *δ* = 133.24 (s, *C*H=CHCH_2_), 125.32 (d, *J* = 5.9 Hz, CH=*C*HCH_2_), 127.63–118.60 (m, *J* = 287.3 Hz, *C*F_3_), 109.35 (s, *C*(CH_3_)_2_), 95.75–92.17 (m, *C*F), 76.52 (s, *C*HCH=CH), 69.26 (s, O*C*H_2_), 67.64 (dd, *J* = 163.9, 29.3 Hz, *C*P), 63.86 (d, *J* = 4.2 Hz, 2 × O*C*H_2_CH_3_), 63.40 (d, *J* = 7.2 Hz, O*C*H_2_CH_3_), 32.63 (d, *J* = 20.4 Hz, *C*H_2_CF), 26.57 (s, *C*H_3_), 25.81 (s, *C*H_3_), 16.30 (“t”, *J* = 5.8 Hz, 2 × OCH_2_*C*H_3_). ^31^P NMR (162 MHz, Chloroform-*d*) *δ* = 18.41–18.20 (m). ^19^F NMR (376 MHz, Chloroform-*d*) *δ* = −76.49–−76.61 (m, C*F*_3_), −174.27 (ddh, *J* = 27.8, 13.4, 6.7 Hz, *F*). ^19^F{^1^/H} NMR (376 MHz, Chloroform-*d*) *δ* = −76.53 (dd, *J* = 6.5, 3.3 Hz, C*F*_3_), −174.26 (p, *J* = 6.6 Hz, *F*). Minor: ^1^H NMR (400 MHz, Chloroform-*d*) *δ* = 5.83–5.77 (m, 1H, CH=C*H*CH_2_), 5.58 (d, *J* = 6.8 Hz, 1H, C*H*=CHCH_2_), 4.67 (td, *J* = 7.3, 1H, O*H*), 4.44 (q, *J* = 7.1 Hz, 1H, C*H*CH=CH), 4.36–4.25 (m, 1H, C*H*P), 4.22–4.06 (m, 4H, 2 × OC*H*_2_CH_3_), 4.01 (dd, *J* = 8.2, 6.2 Hz, 1H, OC*H*H), 3.50 (dd, *J* = 7.9, 2.3 Hz, 1H, OCH*H*), 3.08–2.92 (m, 1H, CH*H*CF), 2.92–2.74 (m, 2H), 1.35 (s, 3H, C*H*_3_), 1.31 (s, 3H, C*H*_3_), 1.28 (t, *J* = 7.0 Hz, 6H, 2 × OCH_2_C*H*_3_). ^13^C NMR (101 MHz, Chloroform-*d*) *δ* = 133.18 (s, *C*H=CHCH_2_), 125.32 (d, *J* = 5.9 Hz, CH=*C*HCH_2_), 127.63–118.60 (m, *J* = 287.3 Hz, *C*F_3_), 109.33 (s, *C*(CH_3_)_2_), 95.75–92.17 (m, *C*F), 76.59 (s, *C*HCH=CH), 69.22 (s, O*C*H_2_), 67.61 (dd, *J* = 163.0, 28.9 Hz, *C*P), 63.78 (d, *J* = 4.4 Hz, O*C*H_2_CH_3_), 63.38 (d, *J* = 7.3 Hz, O*C*H_2_CH_3_), 32.59 (d, *J* = 20.9 Hz, *C*H_2_CF), 26.58 (s, *C*H_3_), 25.81 (s, *C*H_3_), 16.30 (t, *J* = 5.8 Hz, 2 × OCH_2_*C*H_3_). ^31^P NMR (162 MHz, Chloroform-*d*) *δ* = 18.41–18.20 (m). ^19^F NMR (376 MHz, Chloroform-*d*) *δ* = −76.41–−76.49 (m, C*F*_3_), −174.95 (dtt, *J* = 28.0, 13.4, 6.7 Hz, *F*). ^19^F{^1^/H} (376 MHz, Chloroform-*d*) *δ* = −76.45 (dd, *J* = 6.4, 3.3 Hz, C*F*_3_), −174.94 (p, *J* = 6.7 Hz, *F*). HRMS (ESI) calcd. for C_15_H_25_F_4_O_6_PNa ([M + Na]^+^): 431.1223, found: 431.1226.

Diethyl ((*E*)-5-((*S*)-2,2-dimethyl-1,3-dioxolan-4-yl)-1-hydroxy-2-(trifluoromethyl)pent-4-en-1-yl)phosphonate (**21b/21b′**): Colorless oil (54 mg, 69%, ratio 1:1): Major/Minor: ^1^H NMR (400 MHz, Chloroform-*d*) *δ =* 5.95–5.82 (m, 1H, CH=C*H*CH_2_), 5.65–5.55 (C*H*=CHCH_2_), 4.50 (q, *J* = 8.0, 7.5 Hz, 1H, C*H*CH=CH), 4.36 (dd, *J* = 13.1, 6.9 Hz, 1H, C*H*P), 4.28–4.16 (m, 4H, 4H, 2 × OC*H*_2_CH_3_), 4.09 (ddd, *J* = 7.8, 5.4, 1.4 Hz, 1H, OC*H*H), 3.58 (td, *J* = 7.9, 3.9 Hz, 1H, OCH*H*), 2.80–2.68 (m, 1H, C*H*HCHCF_3_), 2.68–2.57 (m, 1H, C*H*HCHCF_3_), 2.56–2.45 (m, 1H, C*H*CF_3_), 1.44 (s, 3H, C*H*_3_), 1.40 (s, 3H, C*H*_3_), 1.39–1.34 (m, 6H, 2 × OCH_2_C*H*_3_). ^31^P NMR (162 MHz, Chloroform-*d*) *δ =* 21.41 (s). Major: ^13^C NMR (101 MHz, Chloroform-*d*) *δ* = 131.59 (s, *C*H=CHCH_2_), 130.23 (s, CH=*C*HCH_2_), 129.79–128.38 (m, *C*F_3_), 109.22 (s, *C*(CH_3_)_2_), 76.81 (s, *C*HCH=CH), 69.35 (s, O*C*H_2_), 65.30 (dd, *J* = 167.5, 2.8 Hz, *C*P), 63.20 (d, *J* = 2.4 Hz, O*C*H_2_CH_3_), 63.13 (d, *J* = 2.4 Hz, O*C*H_2_CH_3_), 45.13–43.97 (m, *C*CF_3_), 26.64 (s, *C*H_3_), 26.13 (s, *C*H_2_CHCF_3_), 25.85 (s, *C*H_3_), 16.47 (s, OCH_2_*C*H_3_), 16.42 (s, OCH_2_*C*H_3_). ^19^F NMR (376 MHz, Chloroform-*d*) *δ* = −67.82 (d, *J* = 3.2 Hz, C*F*_3_). Minor: ^13^C NMR (101 MHz, Chloroform-*d*) *δ* = 131.34 (s, *C*H=CHCH_2_), 130.23 (s, CH=*C*HCH_2_), 129.79–128.38 (m, *C*F_3_), 109.21 (s, *C*(CH_3_)_2_), 76.81 (s, *C*HCH=CH), 69.30 (s, O*C*H_2_), 65.27 (dd, *J* = 167.5, 2.9 Hz, *C*P), 63.40 (d, *J* = 4.5 Hz, O*C*H_2_CH_3_), 63.33 (d, *J* = 4.5 Hz, O*C*H_2_CH_3_), 45.05–44.11 (m, *C*CF_3_), 26.63 (s, *C*H_3_), 25.85 (s, *C*H_3_), 25.82 (s, *C*H_2_CHCF_3_), 16.38 (s, OCH_2_*C*H_3_), 16.36 (s, OCH_2_*C*H_3_). ^19^F NMR (376 MHz, Chloroform-*d*) *δ* = −67.96 (d, *J* = 3.6 Hz, C*F*_3_). HRMS (ESI) calcd. for C_15_H_26_F_3_O_6_PNa ([M + Na]^+^): 413.1317, found: 413.1320.

## Figures and Tables

**Table 1:** Values of known experimental pK_a_, calculated pK_a_ and calculated pK_a_ with included obtained for Equation (1) parameters A and B.

	Exp. pK_a_ [64]	Calc. pK_a_
Formic Acid	3.8	3.7
Trifluoroacetic Acid	0.2	0.8
Dimethyl Malonate	13.5	11.9
Acetone	20.0	19.7
Chloroacetone	16.5	17.9
2-((2,3,3,3-tetrafluoroprop-1-en-1-yl)oxy)but-3-enal 13	-	13.3
*α*-trifluoromethyl-*γ*,*δ*-unsaturated aldehyde 19b	-	15.1

## Data Availability

The data are available from the corresponding author.

## Supplementary Materials

The following supporting information can be downloaded at: https://www.mdpi.com/article/10.3390/molecules27175404/s1, Copies of NMR spectra, DFT coordinates.
