# Supplementary material for: Design and Synthesis of New α-hydroxy β-fluoro/β-trifluoromethyl and Unsaturated Phosphonates from Carbohydrate-Derived Building Blocks via Pudovik and Horner–Wadsworth–Emmons Reactions
Source: Molecules. 2022 Aug 24;27(17):5404. doi: 10.3390/molecules27175404 (PMC9457578; doi:10.3390/molecules27175404)
Supplement: Supplementary file 1 [file molecules-27-05404-s001.zip › molecules-1885520-supplementary.pdf]

Design and synthesis of new α-hydroxy β-fluoro/β-trifluoromethyl and unsaturated phosphonates from carbohydrate-derived building blocks via Pudovik and Horner–Wadsworth–Emmons reactions

Monika Bilska-Markowska [1], Wojciech Jankowski [1], Marcin Hoffmann [1] and Marcin Kaźmierczak [1,2,*]

[1] Faculty of Chemistry, Adam Mickiewicz University in Poznań, Uniwersytetu Poznańskiego 8, 61-614 Poznań, Poland
[2] Centre for Advanced Technologies, Adam Mickiewicz University in Poznań, Uniwersytetu Poznańskiego 10, 61-614 Poznań, Poland
* Correspondence: marcin.kazmierczak@amu.edu.pl

NMR Data……………………………………………………………………………………2

DFT Coordinates……………..…………………………………..…………………………37

NMR Data:

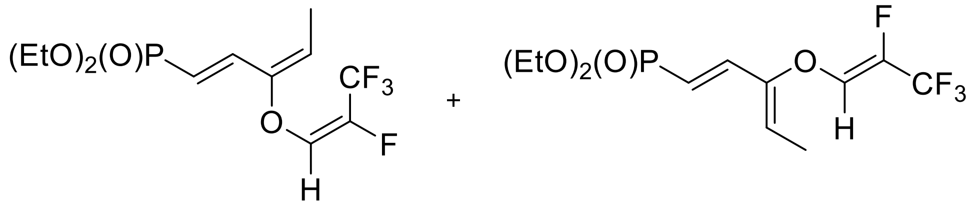

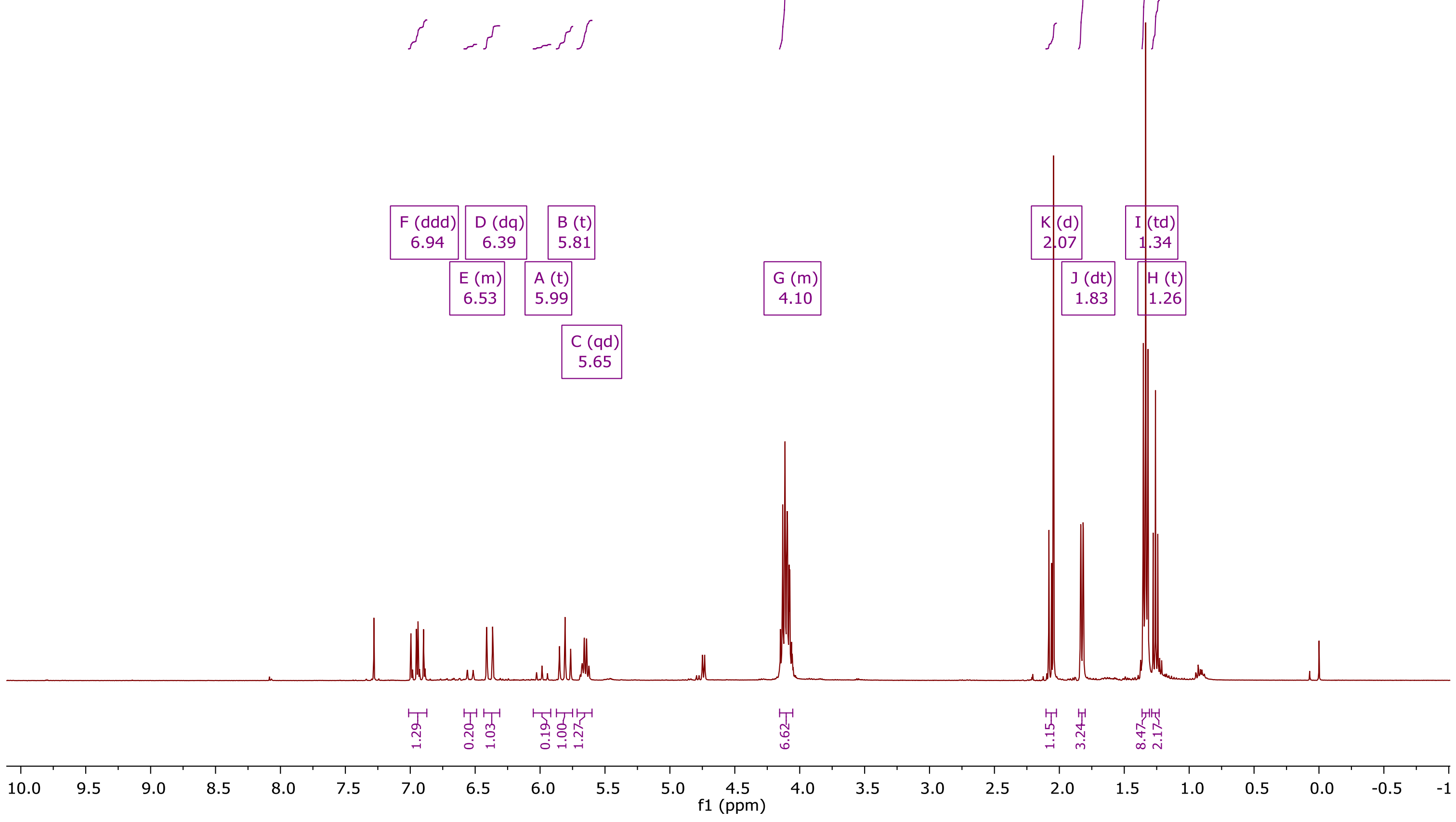

^{1}H NMR of **14/14′**.

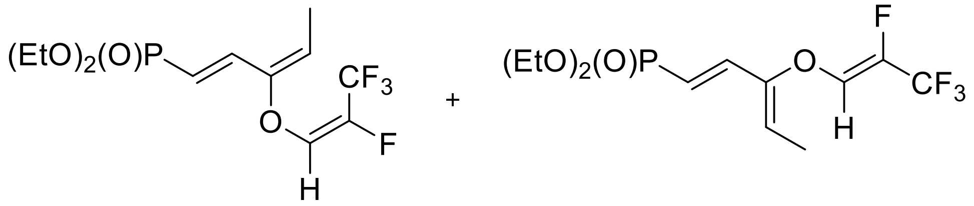

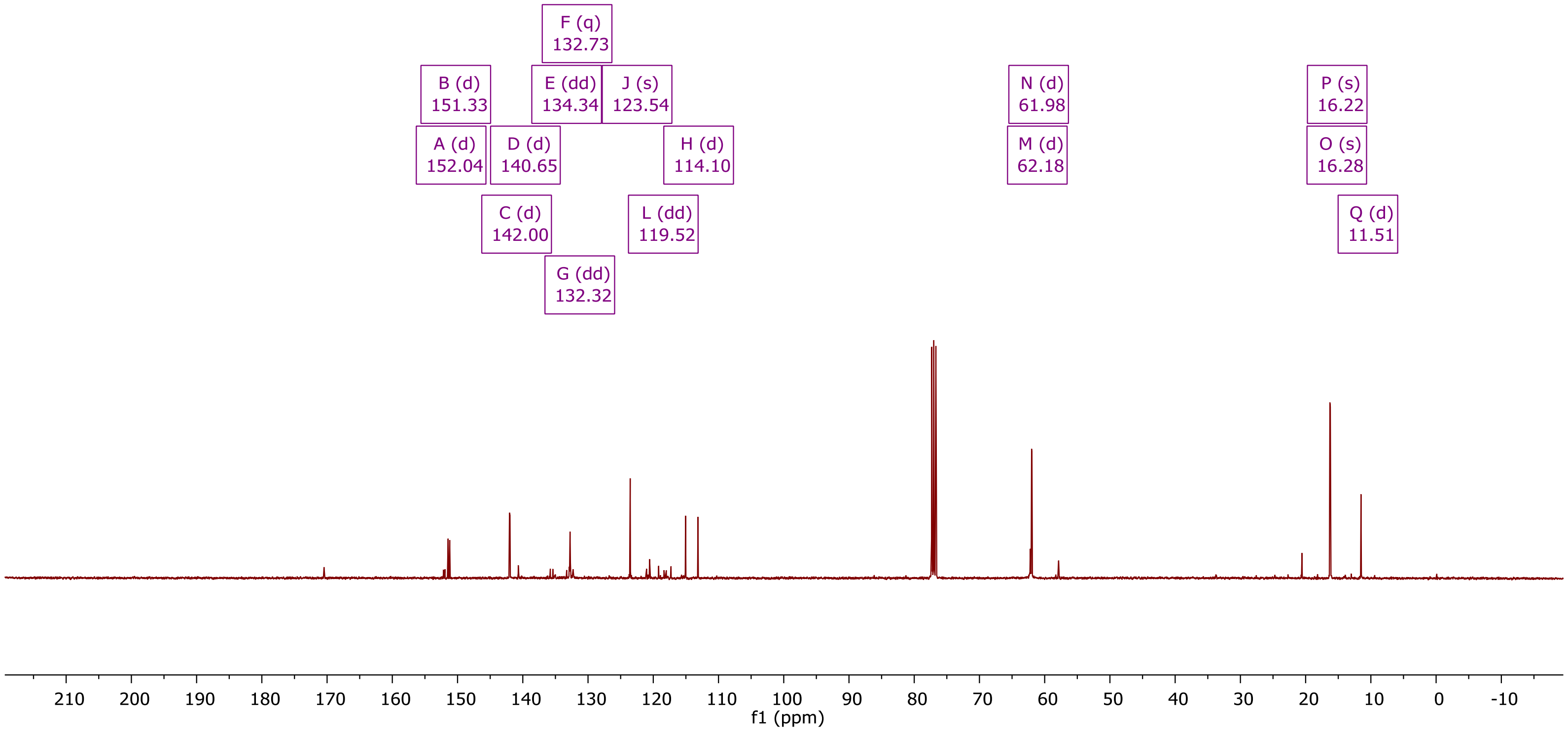

^{13}C NMR of **14/14′**.

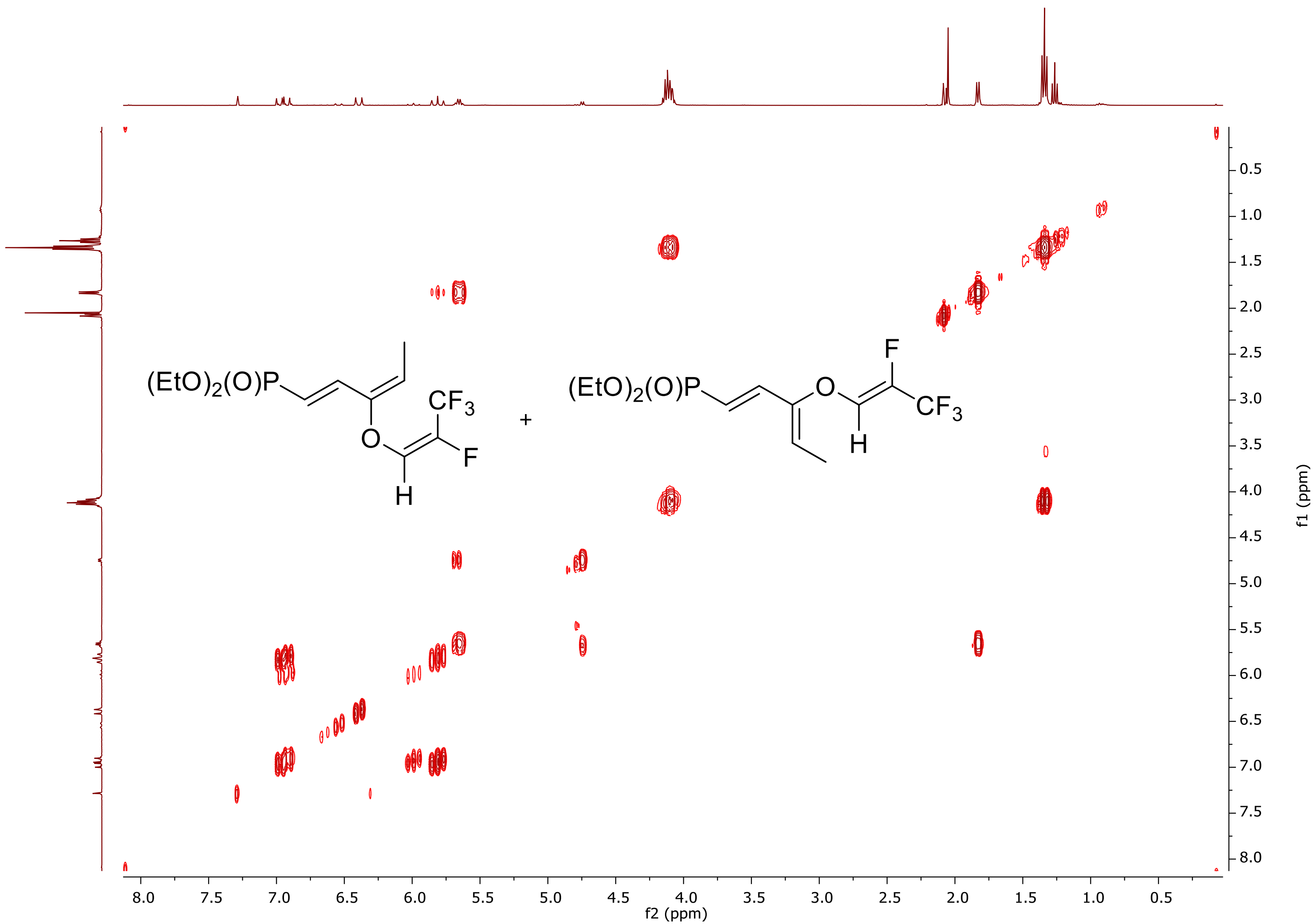

^{1}H-^{1}H COSY **14/14′**.

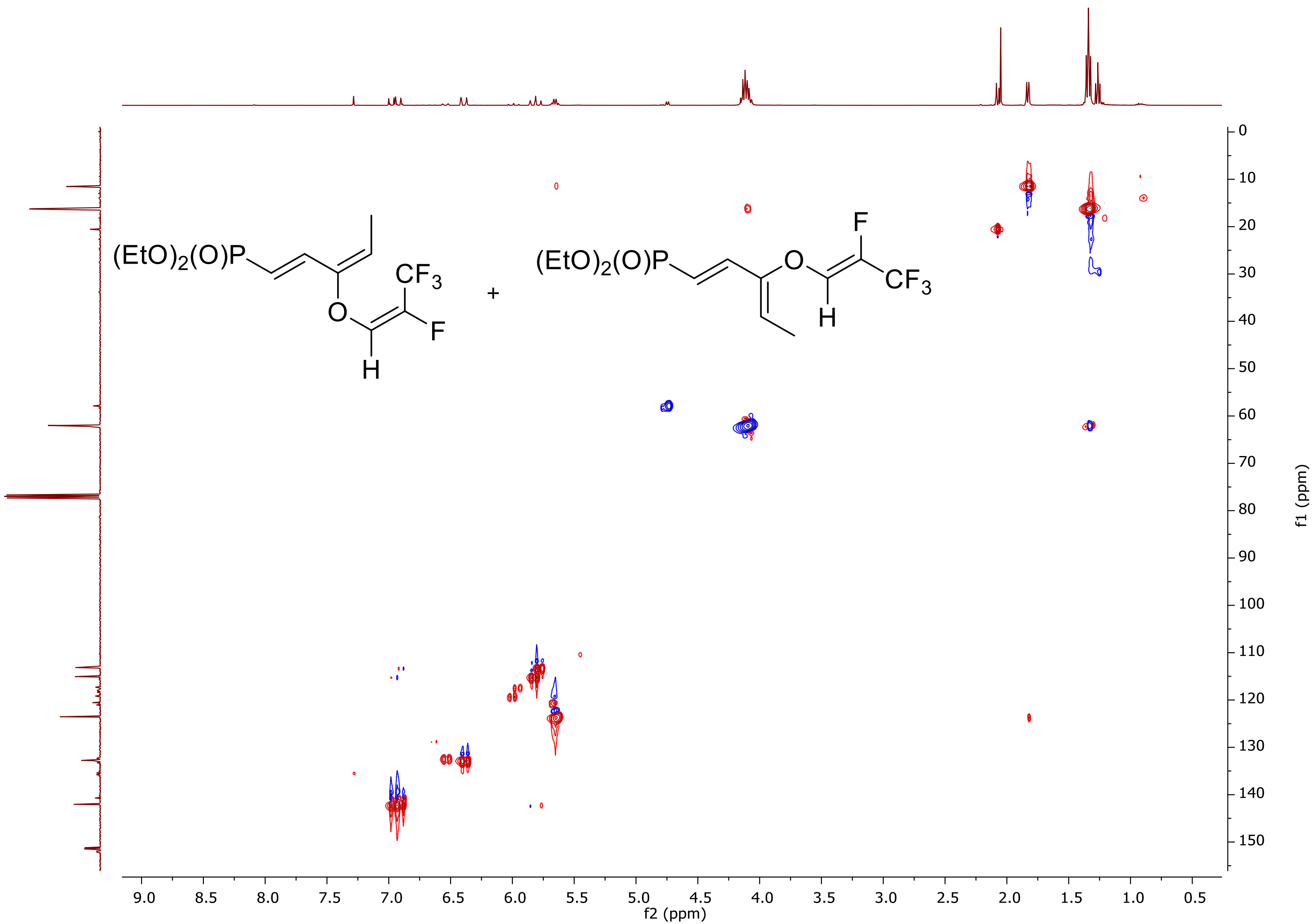

^{1}H-^{13}C HSQC of **14/14′**.

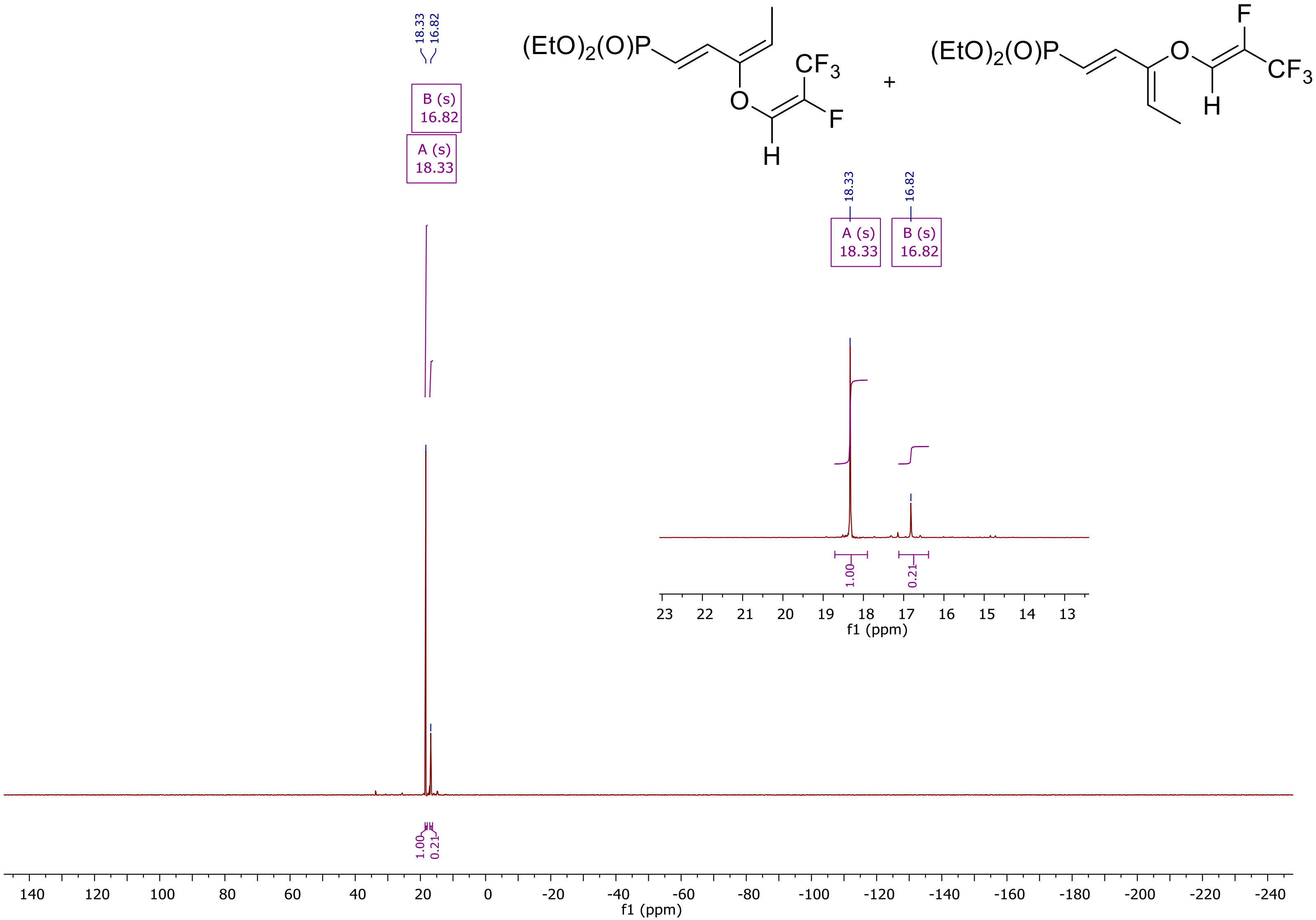

^{31}P{/^{1}H} NMR of **14/14′**.

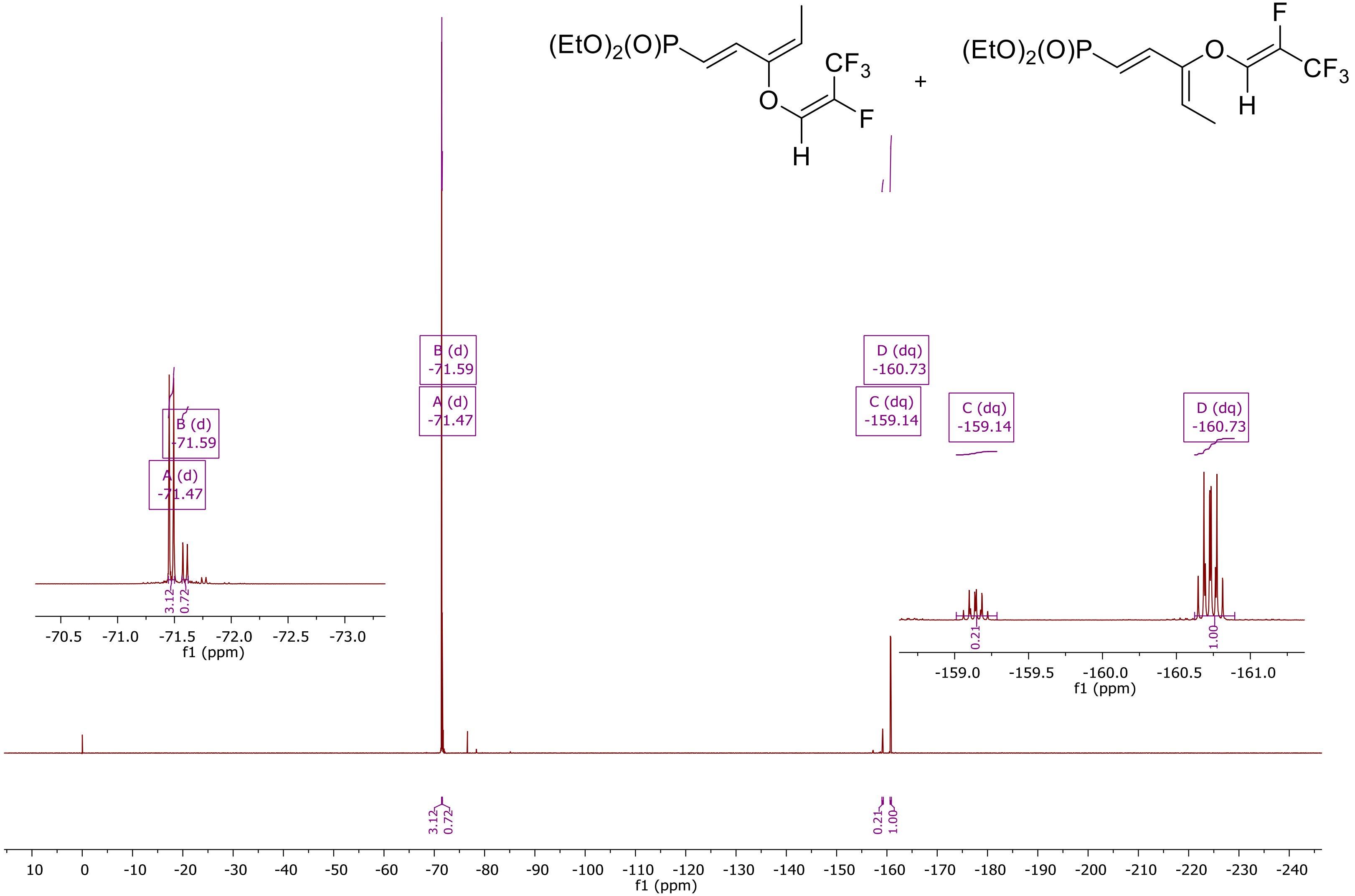

^{19}F NMR of **14/14′**.

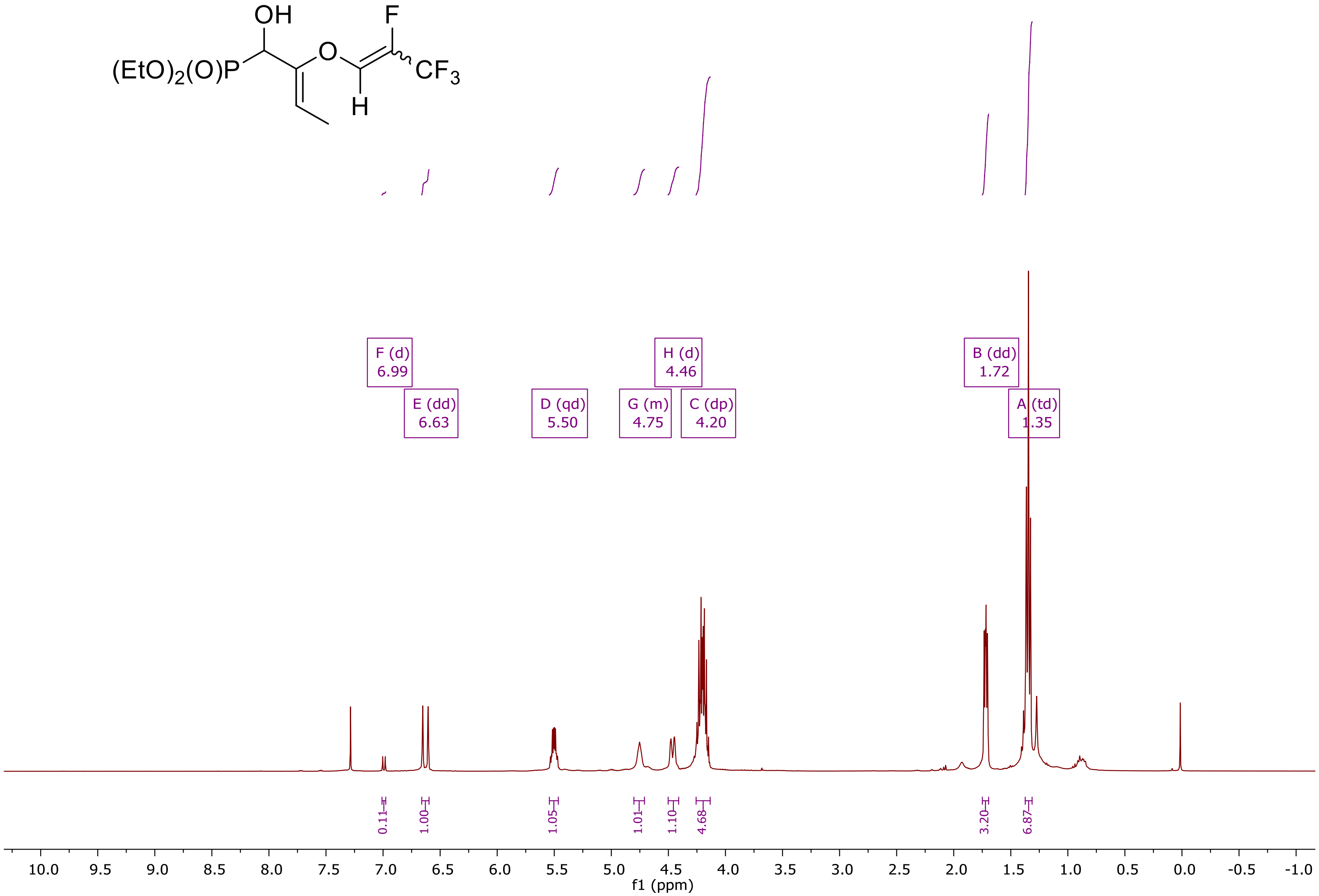

^{1}H NMR of **15/15′**.

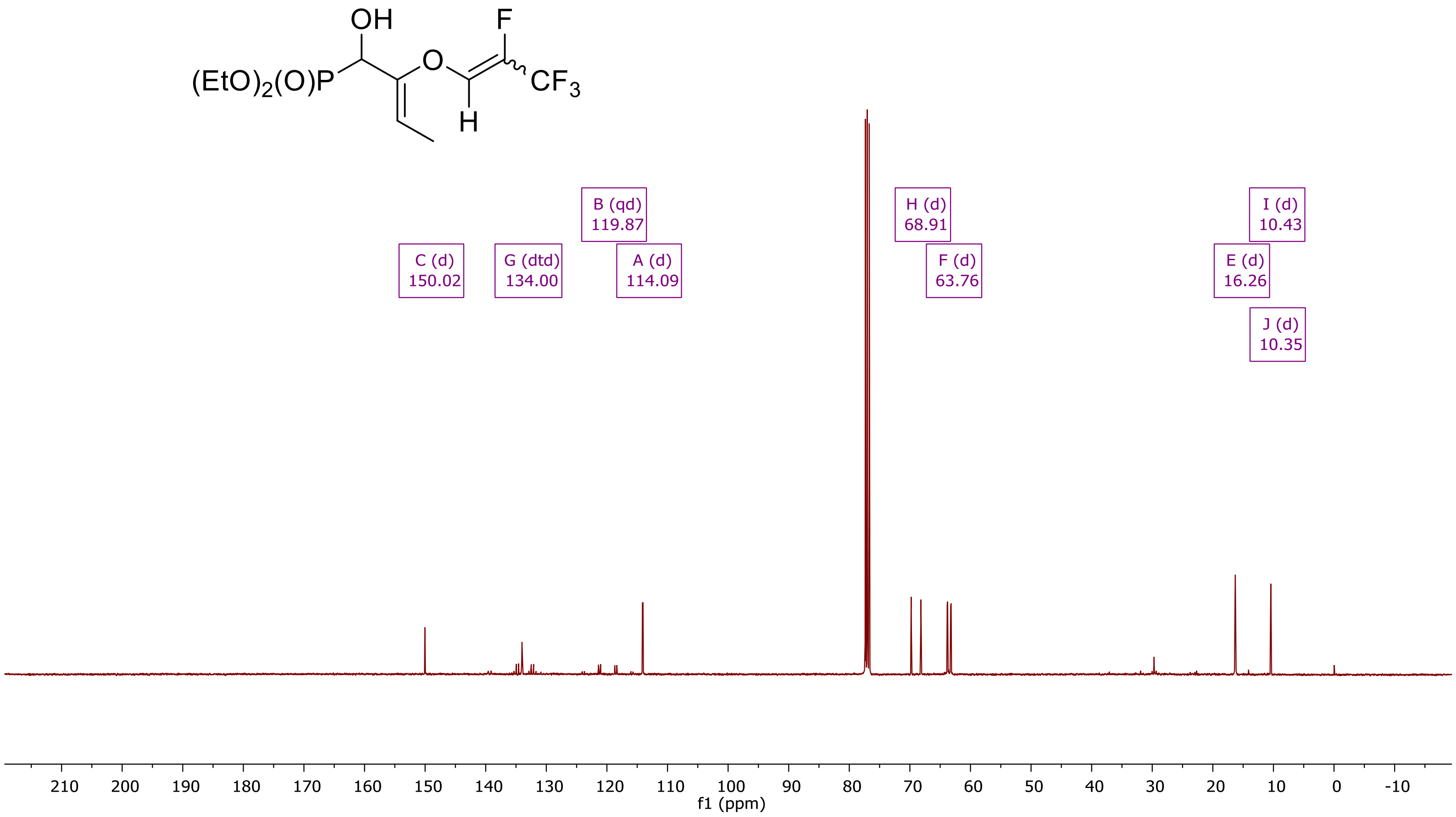

^{13}C NMR of **15/15′**.

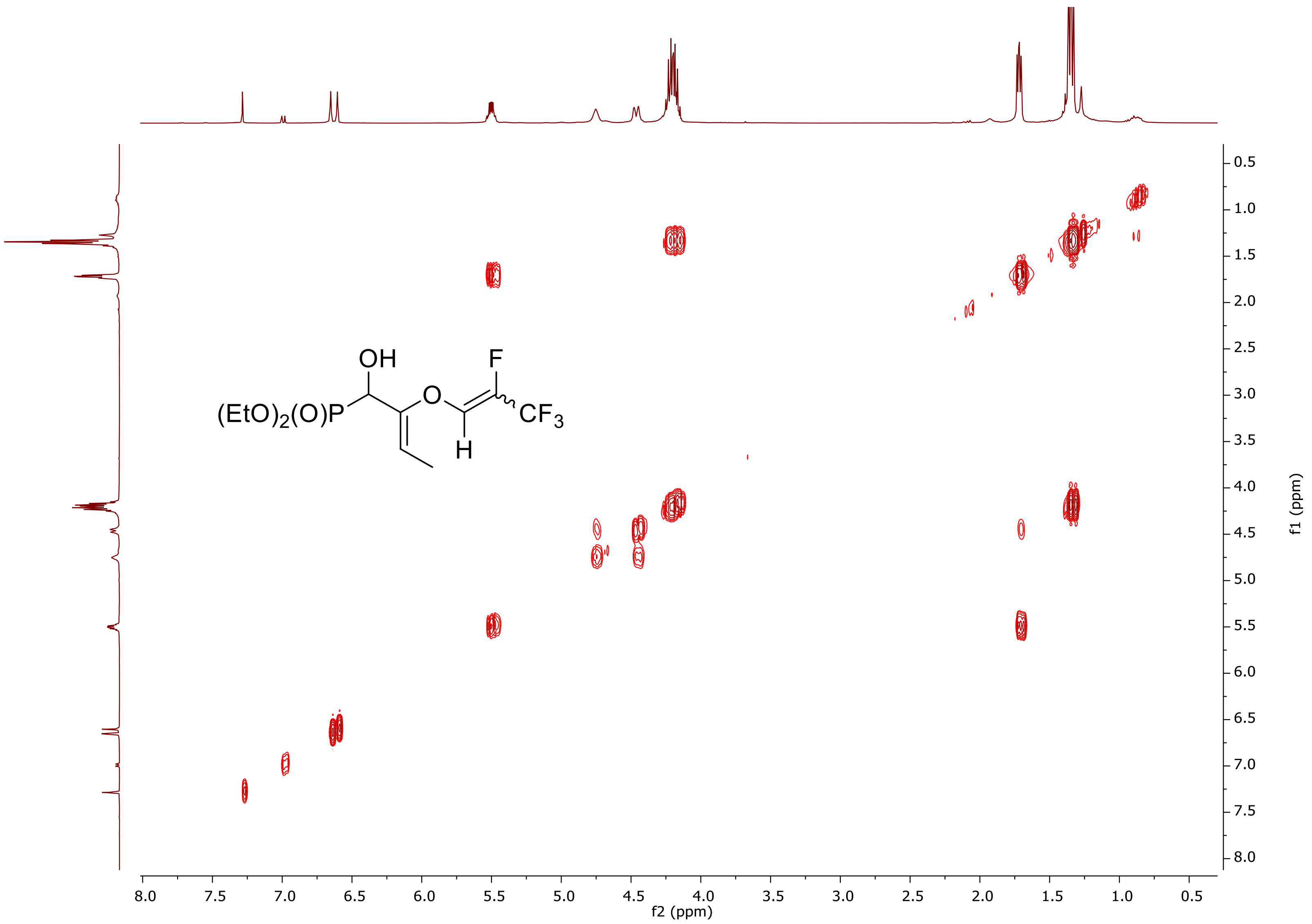

^1H-^1H COSY of **15/15′**.

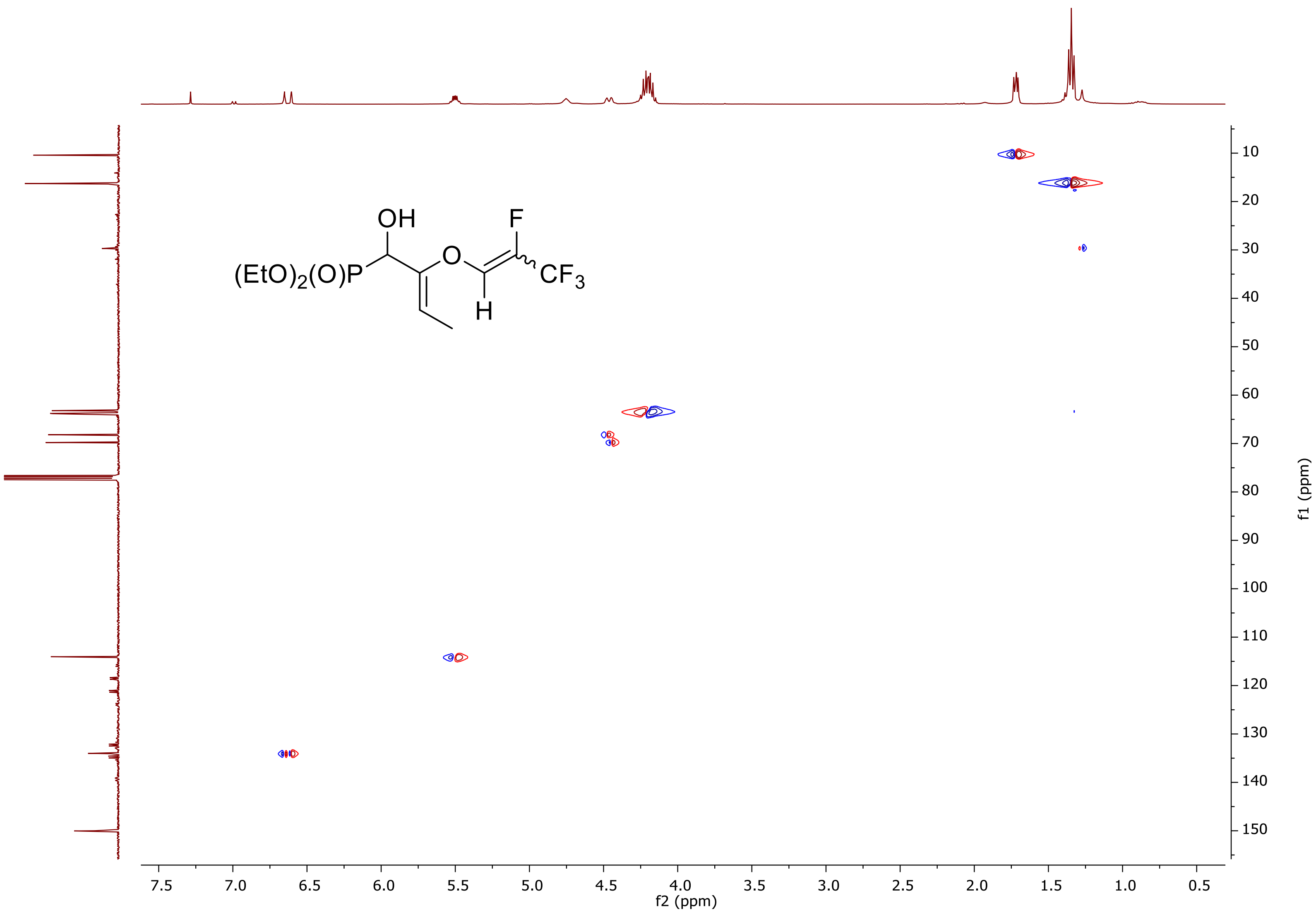

^{1}H-^{13}C HSQC of **15/15′**.

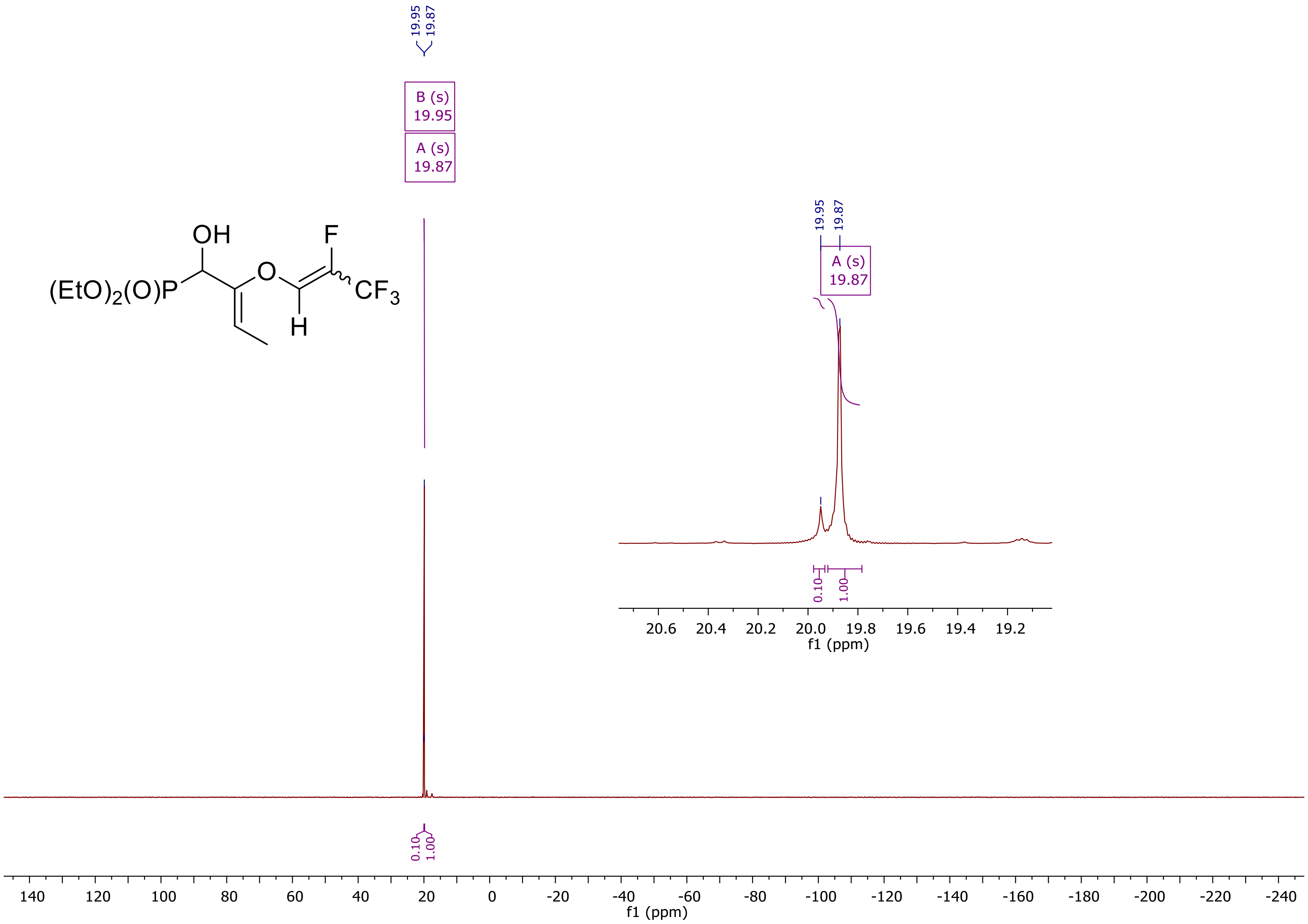

$^{31}P\{/^{1}H\}$ NMR of **15/15′**.

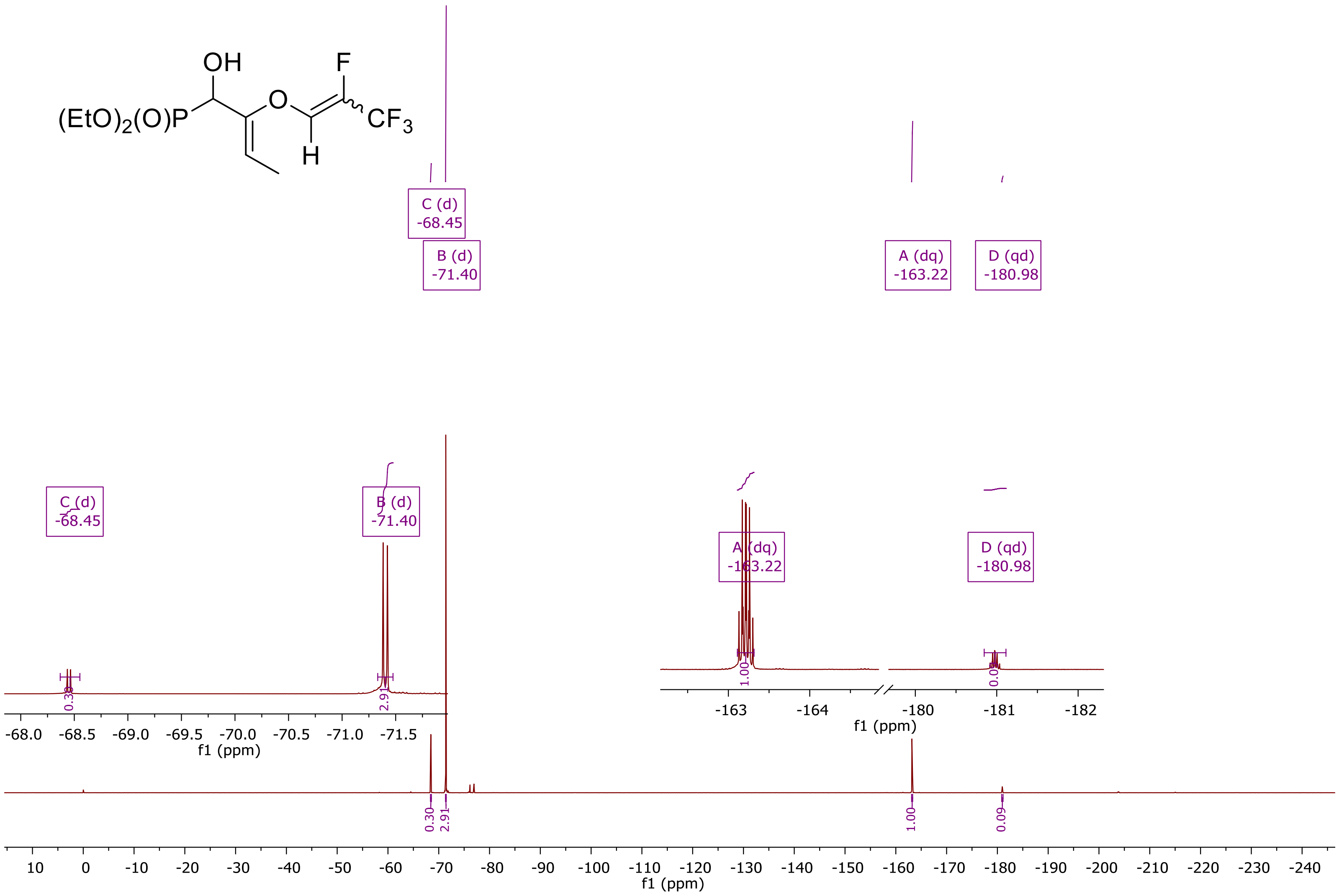

^{19}F NMR of **15/15′**.

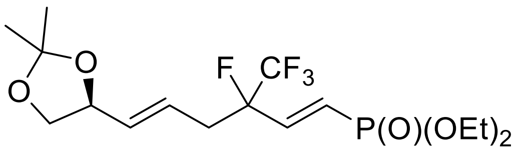

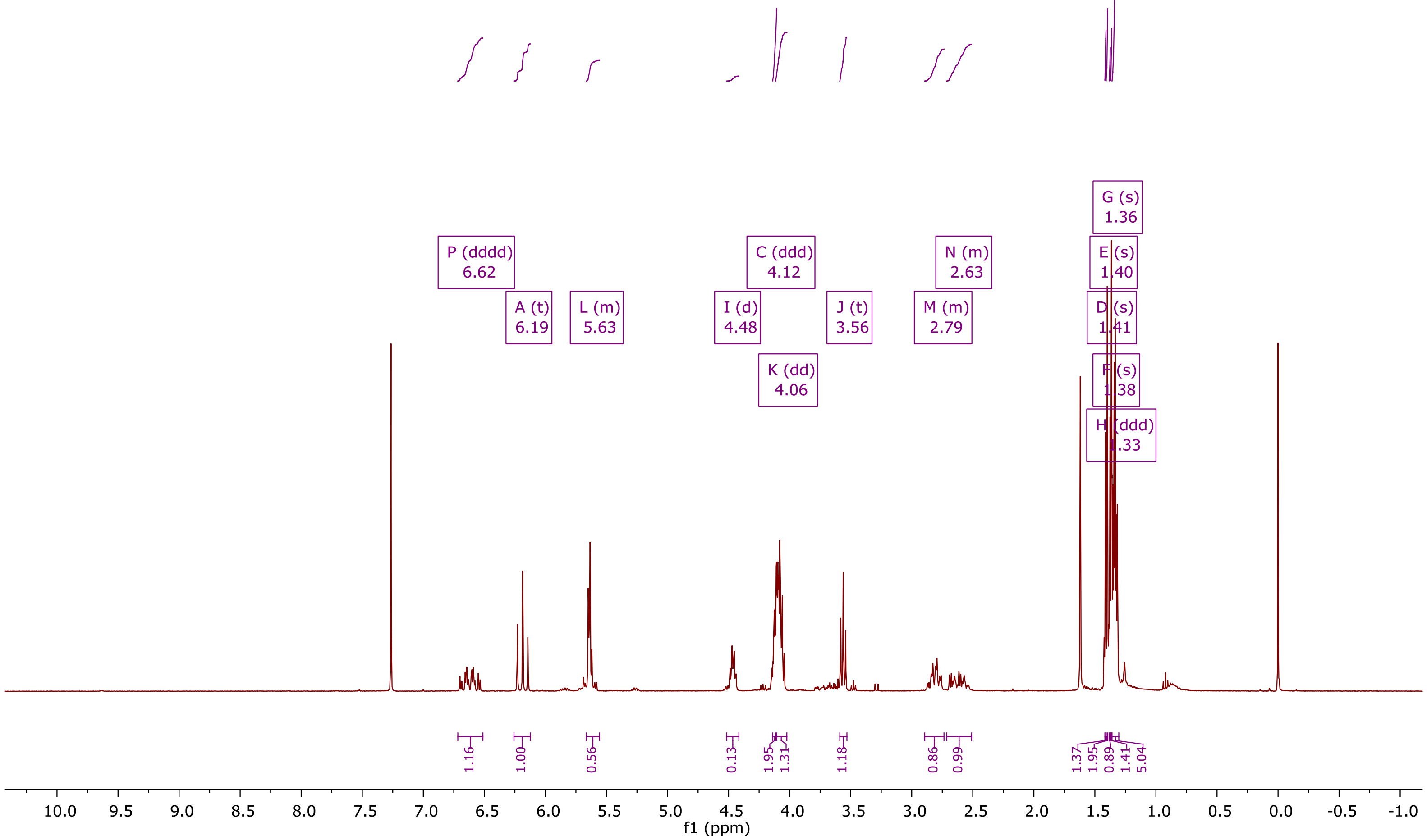

^{1}H NMR of **20a/20a′**.

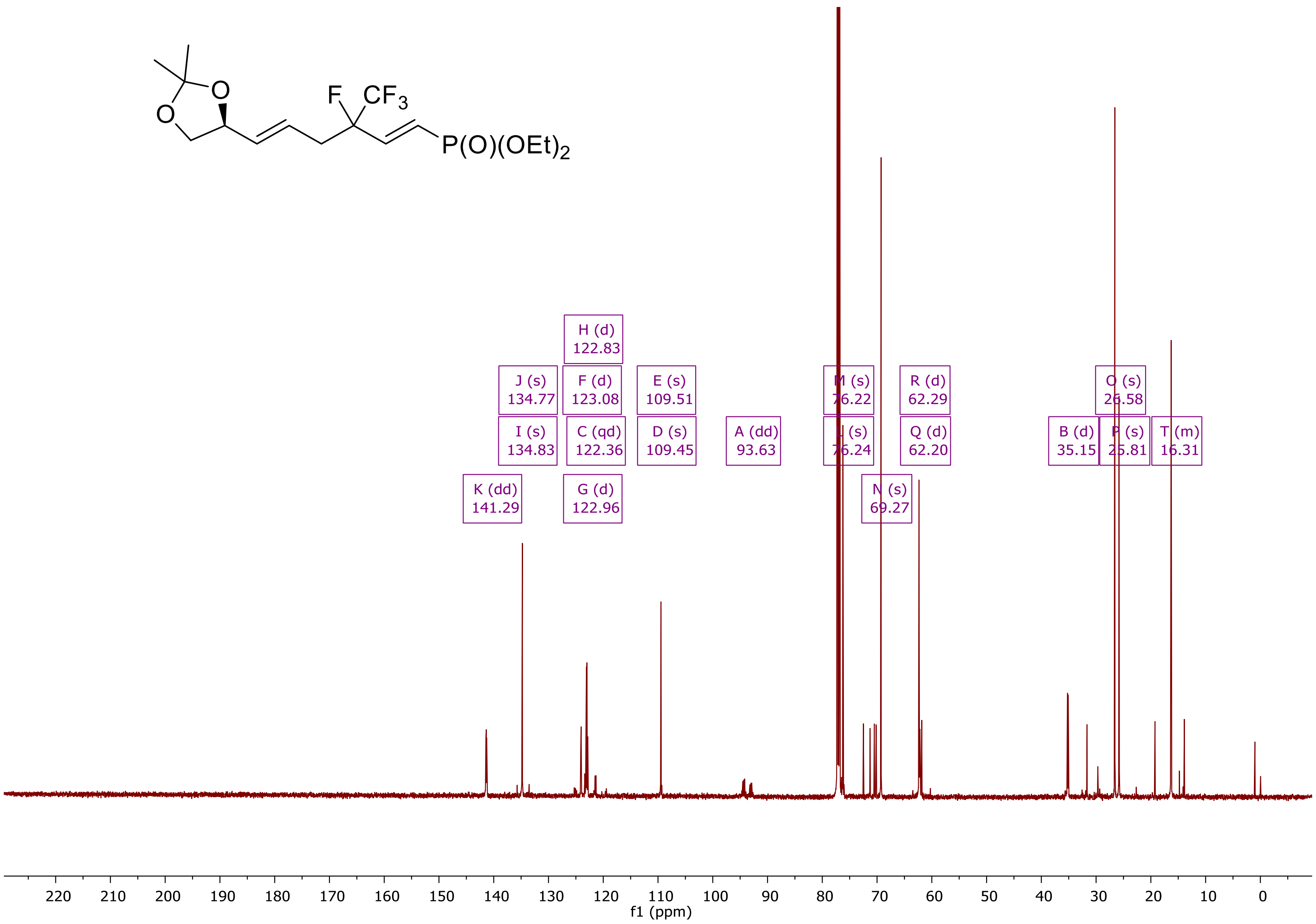

^{13}C NMR of **20a/20a′**.

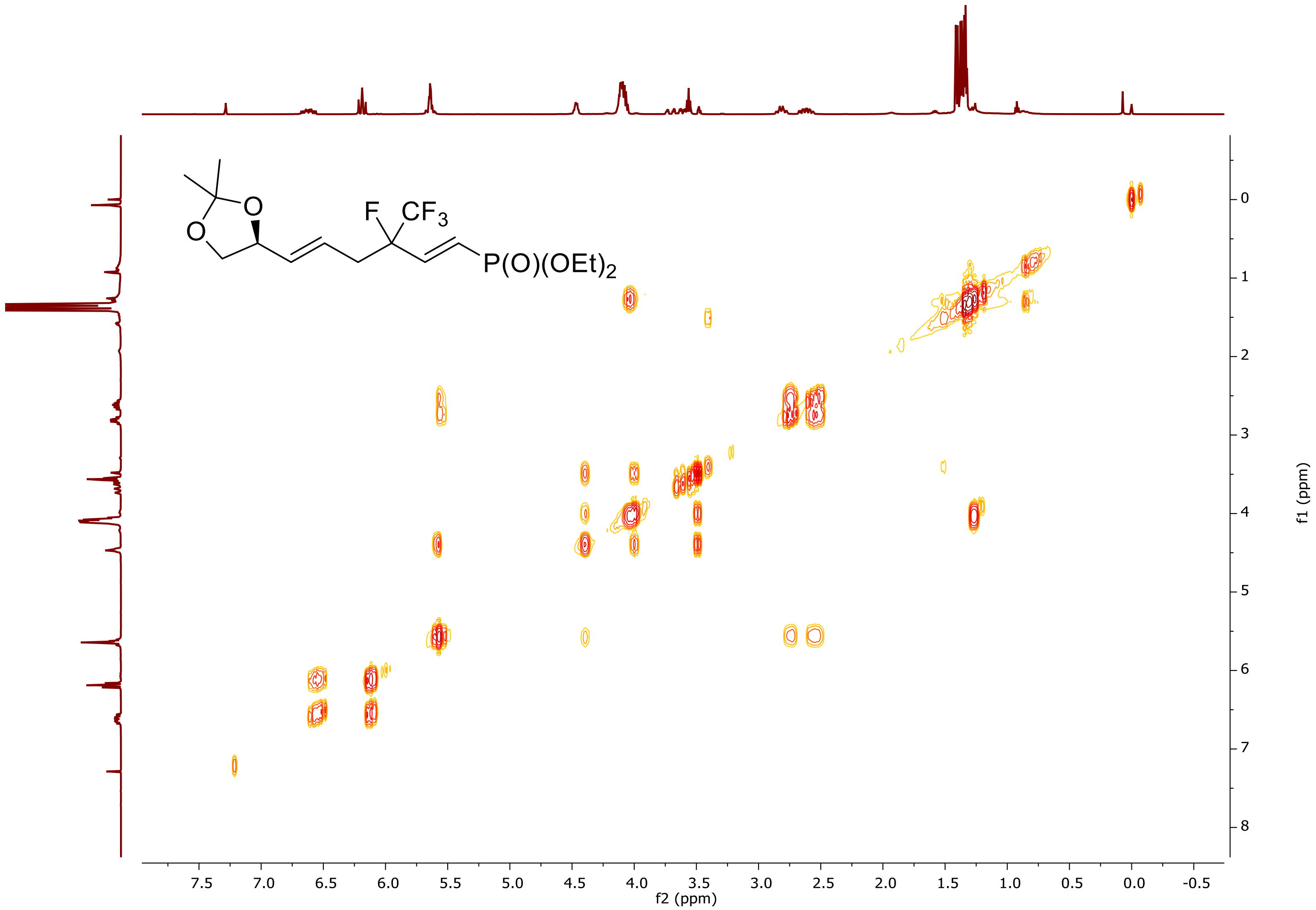

^1H-^1H COSY of **20a/20a′**.

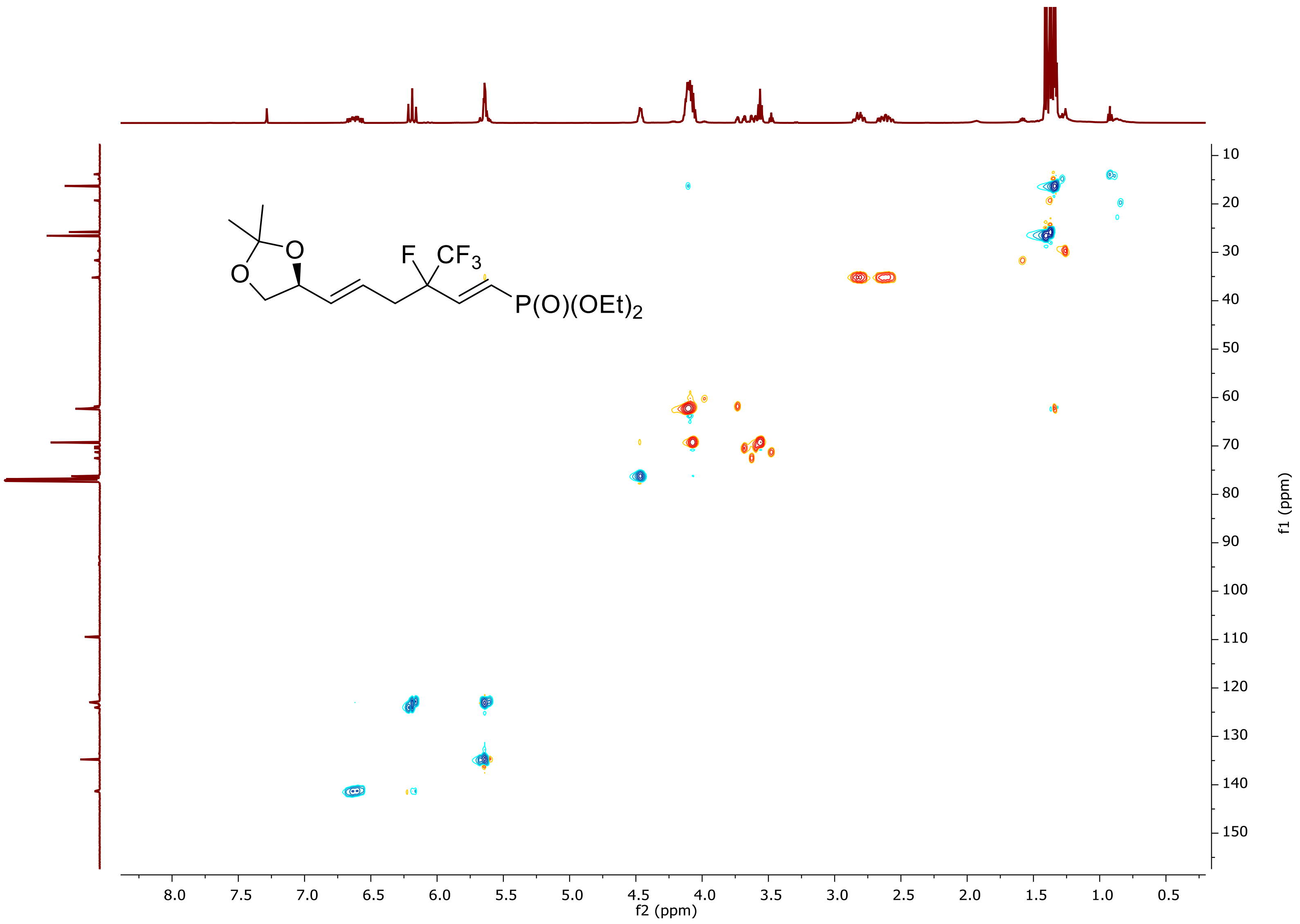

^{1}H-^{13}C HSQC of **20a/20a′**.

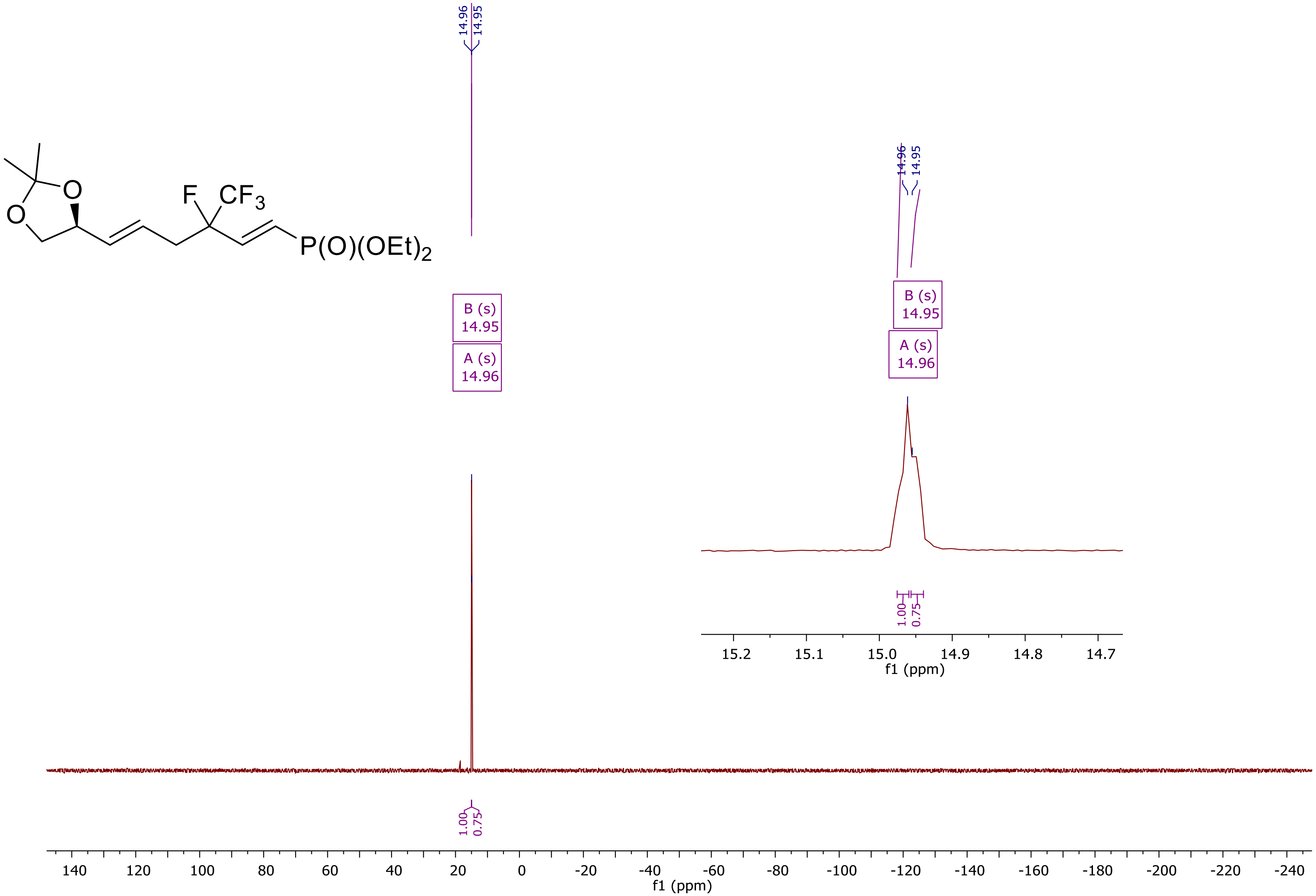

^{31}P{/^{1}H} NMR of **20a/20a′**.

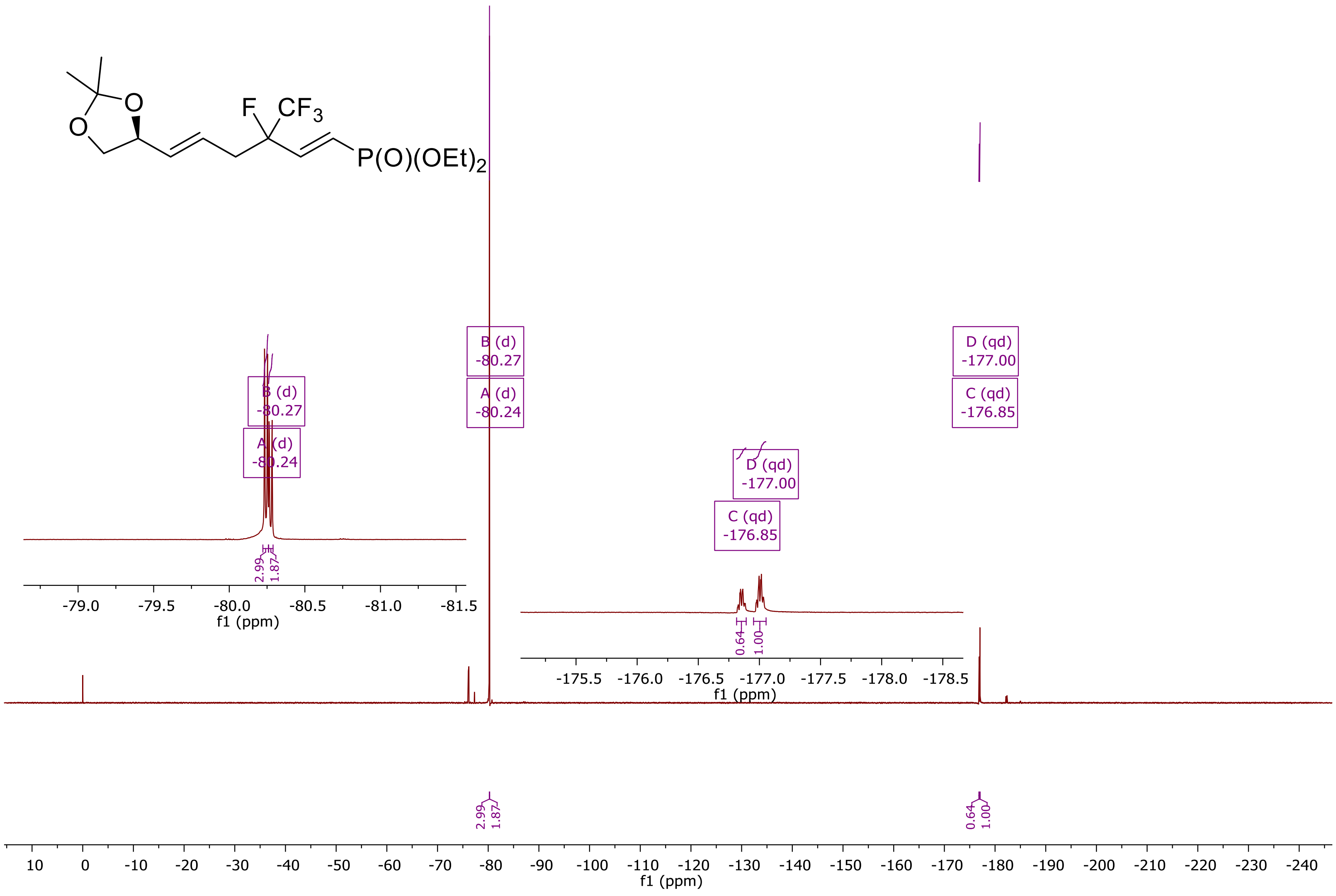

^{19}F NMR of **20a/20a′**.

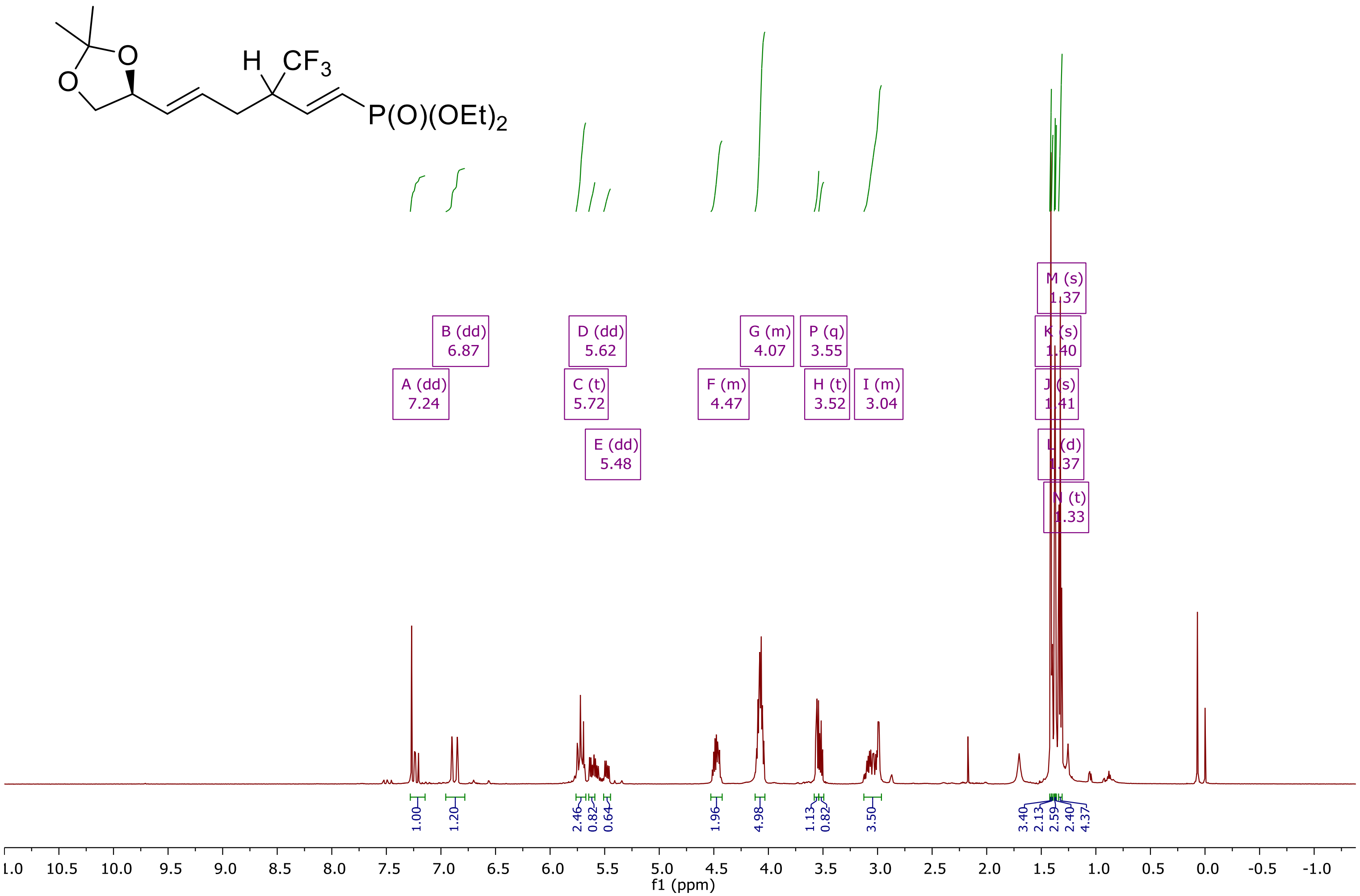

^{1}H NMR of **20b/20b′**.

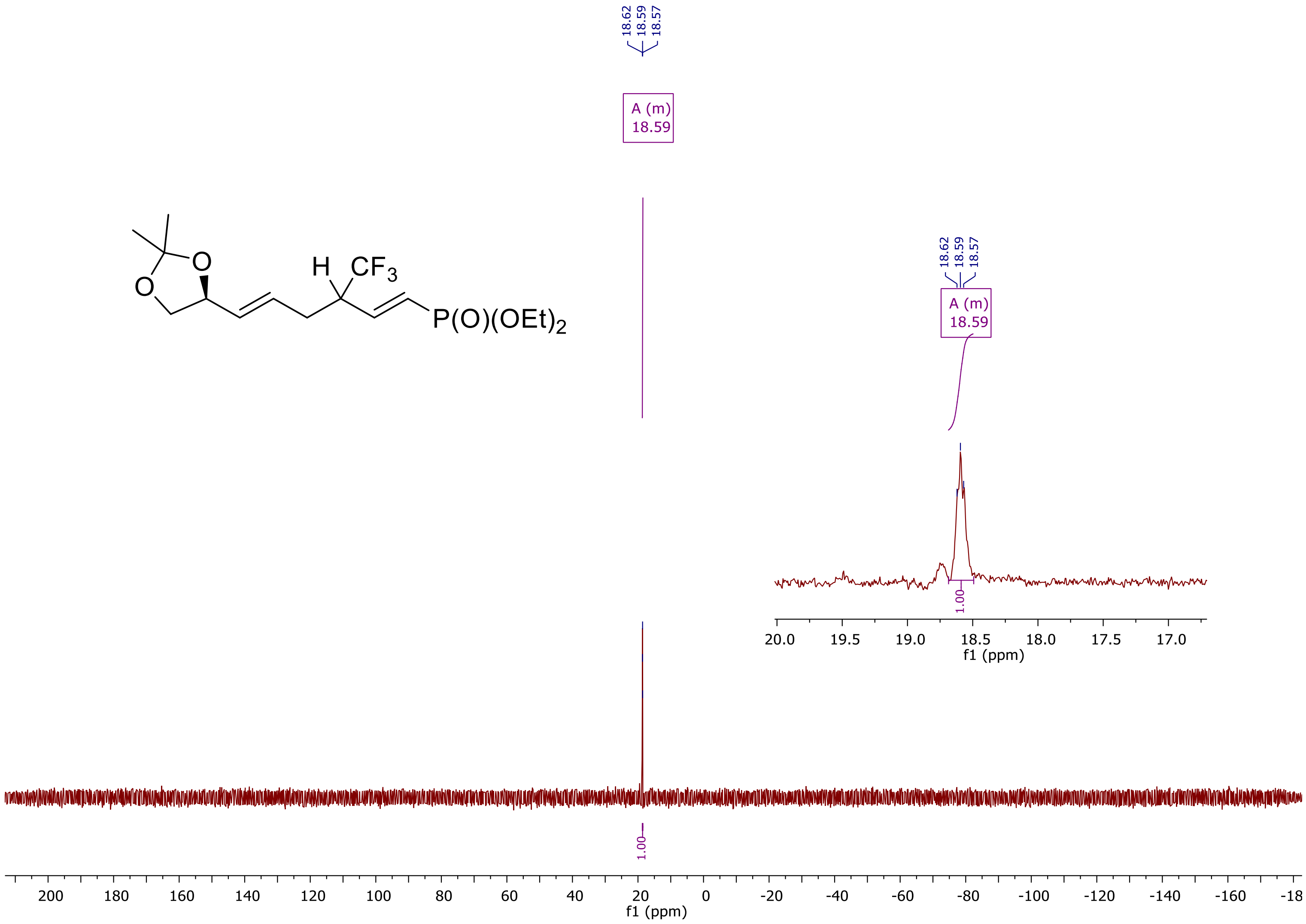

$^{31}P\{/^{1}H\}$ NMR of **20b/20b′**.

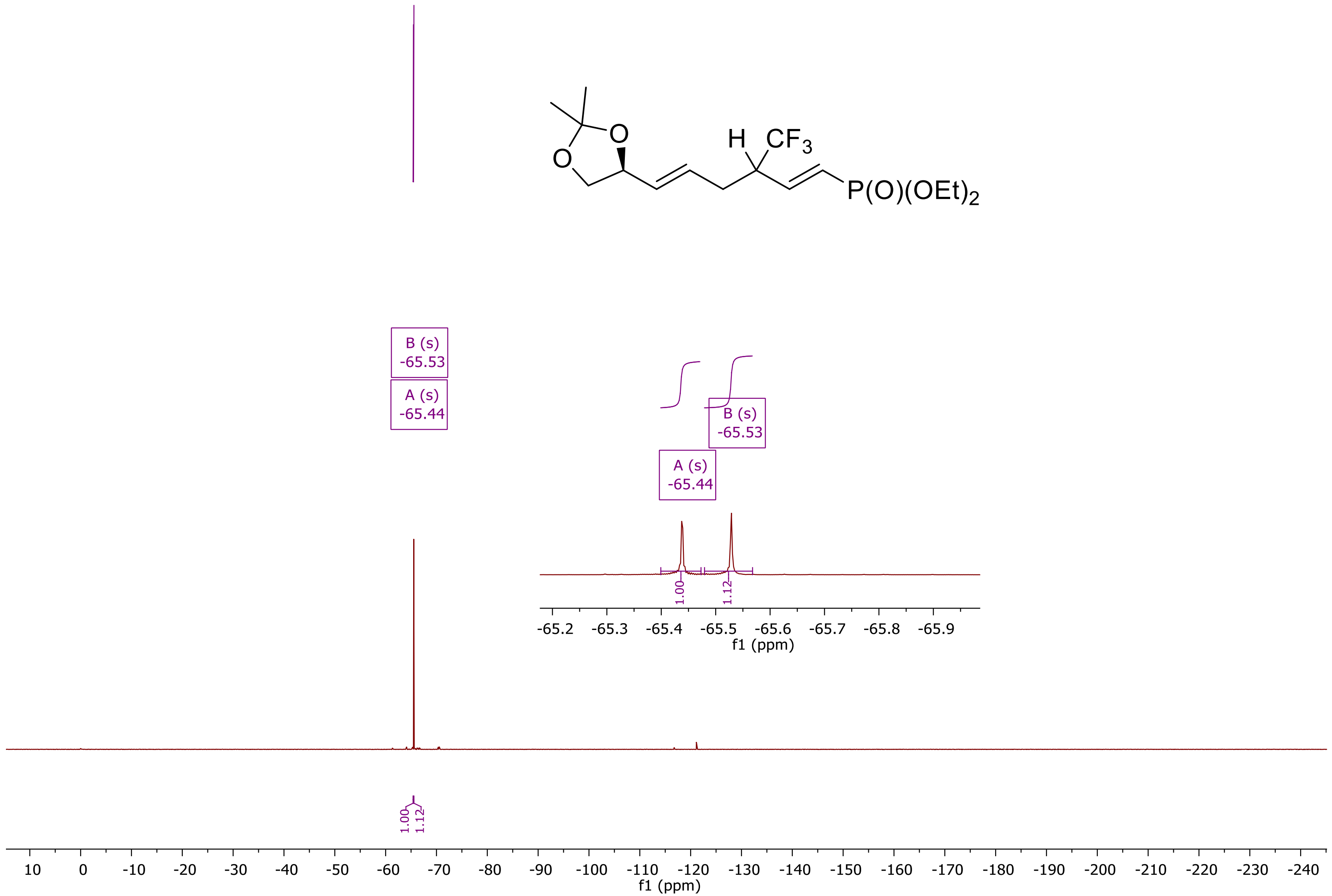

^{19}F NMR of **20b/20b′**.

Display Report

Analysis Info

Analysis Name	D:\Data\Gosia\2022\M. Kazmierczak\22.04.26\MK 2022-38 f 39-46_22.04.26_3.d		
Method	Tune_pos_Standard.m	Operator	malgorzata kasperkowiak
Sample Name	MK 2022-38 f 39-46	Instrument	impact HD 1819696.00156
Comment			

Acquisition Parameter

Source Type	ESI	Ion Polarity	Positive	Set Nebulizer	0.3 Bar
Focus	Active	Set Capillary	4200 V	Set Dry Heater	200 °C
Scan Begin	50 m/z	Set End Plate Offset	-500 V	Set Dry Gas	4.0 l/min
Scan End	500 m/z	Set Charging Voltage	2000 V	Set Divert Valve	Source
		Set Corona	0 nA	Set APCI Heater	0 °C

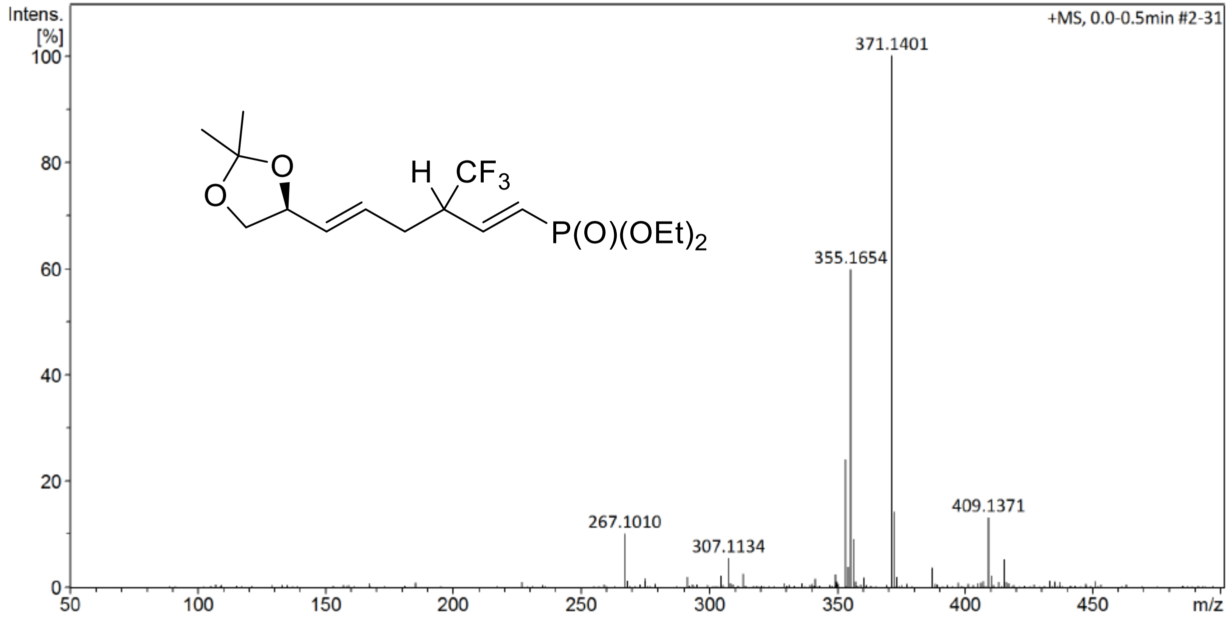

HRMS of **20b/20b′**.

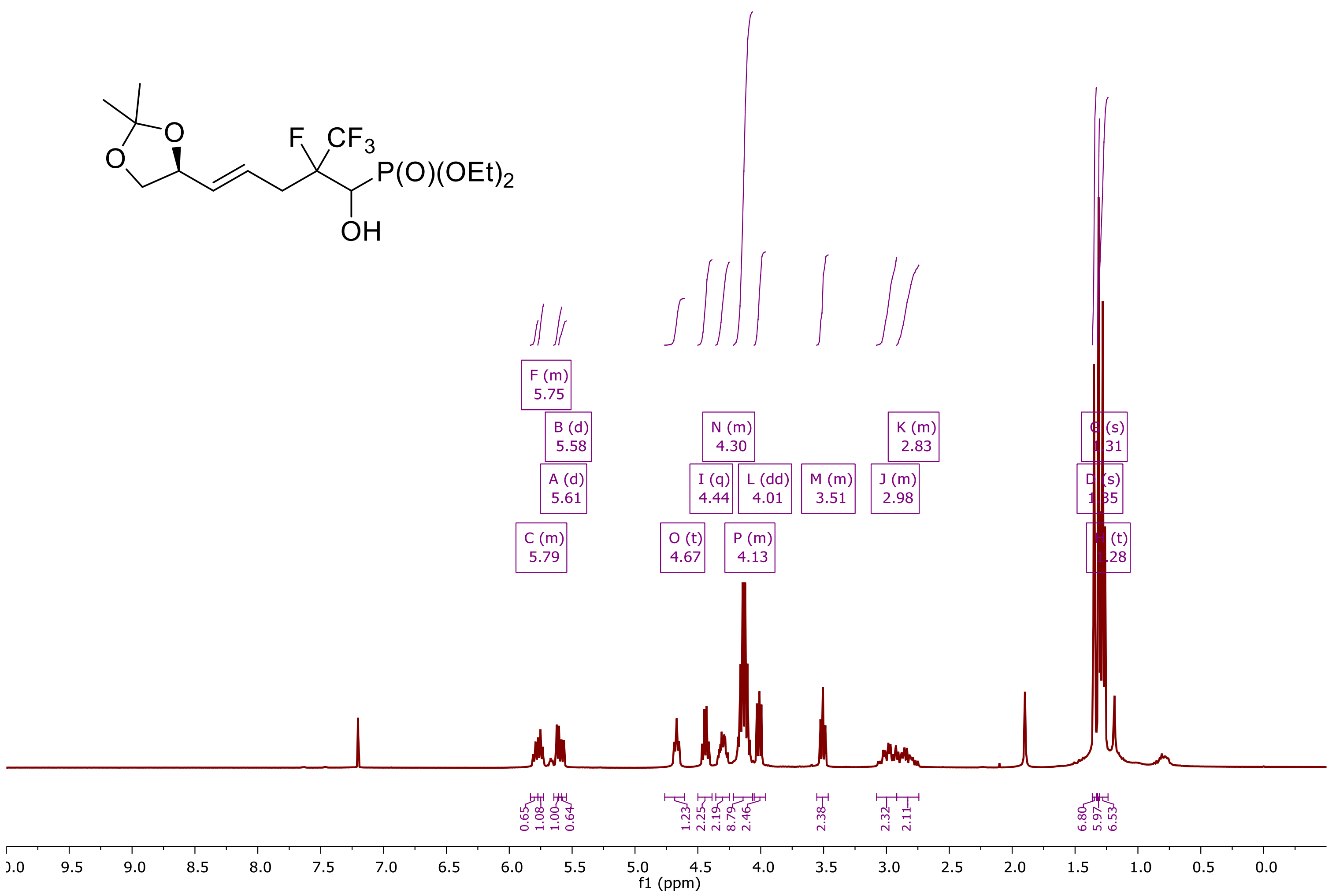

¹H NMR of **21a/21a′**.

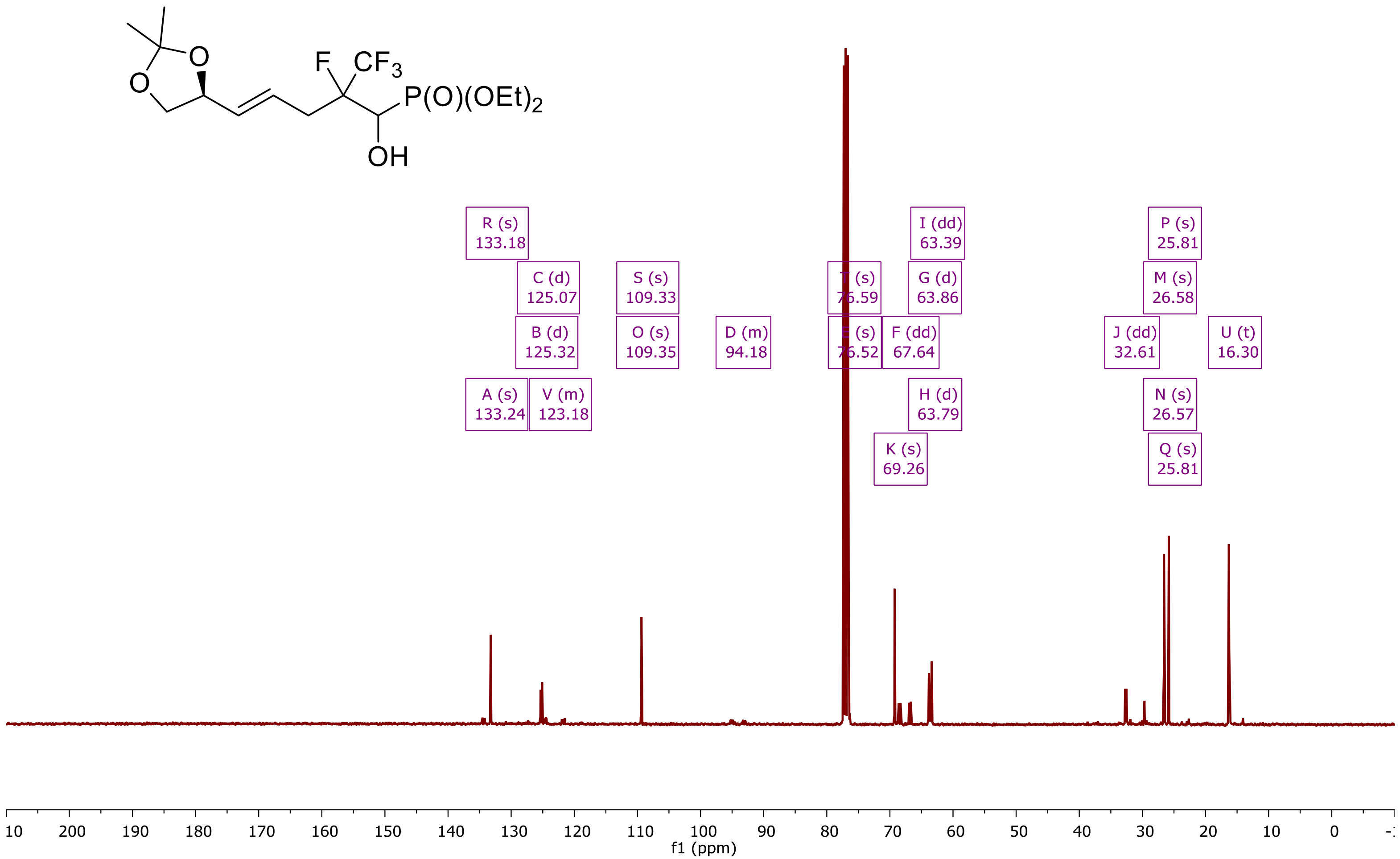

^{13}C NMR of **21a/21a′**.

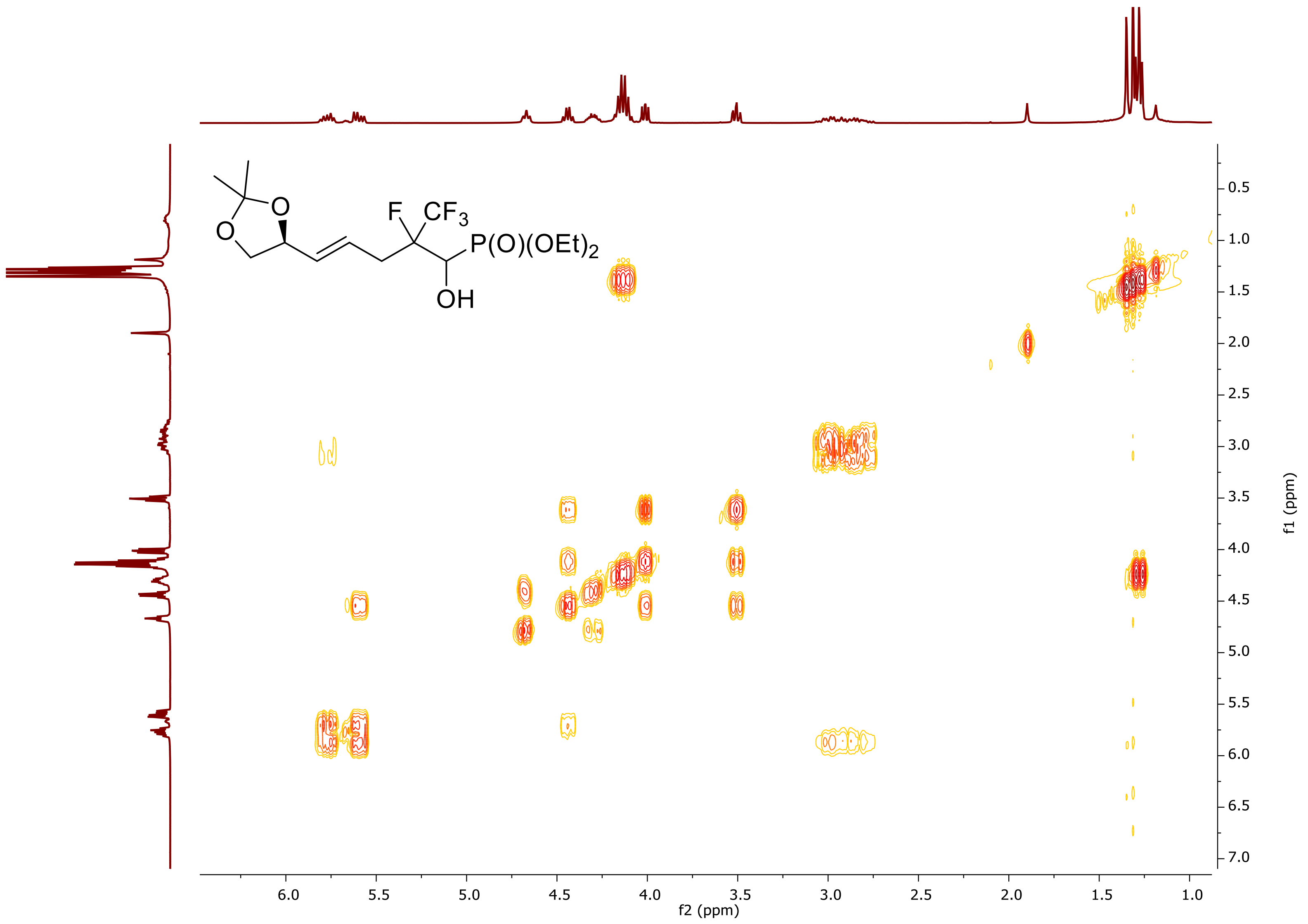

^{1}H-^{1}H COSY of **21a/21a′**.

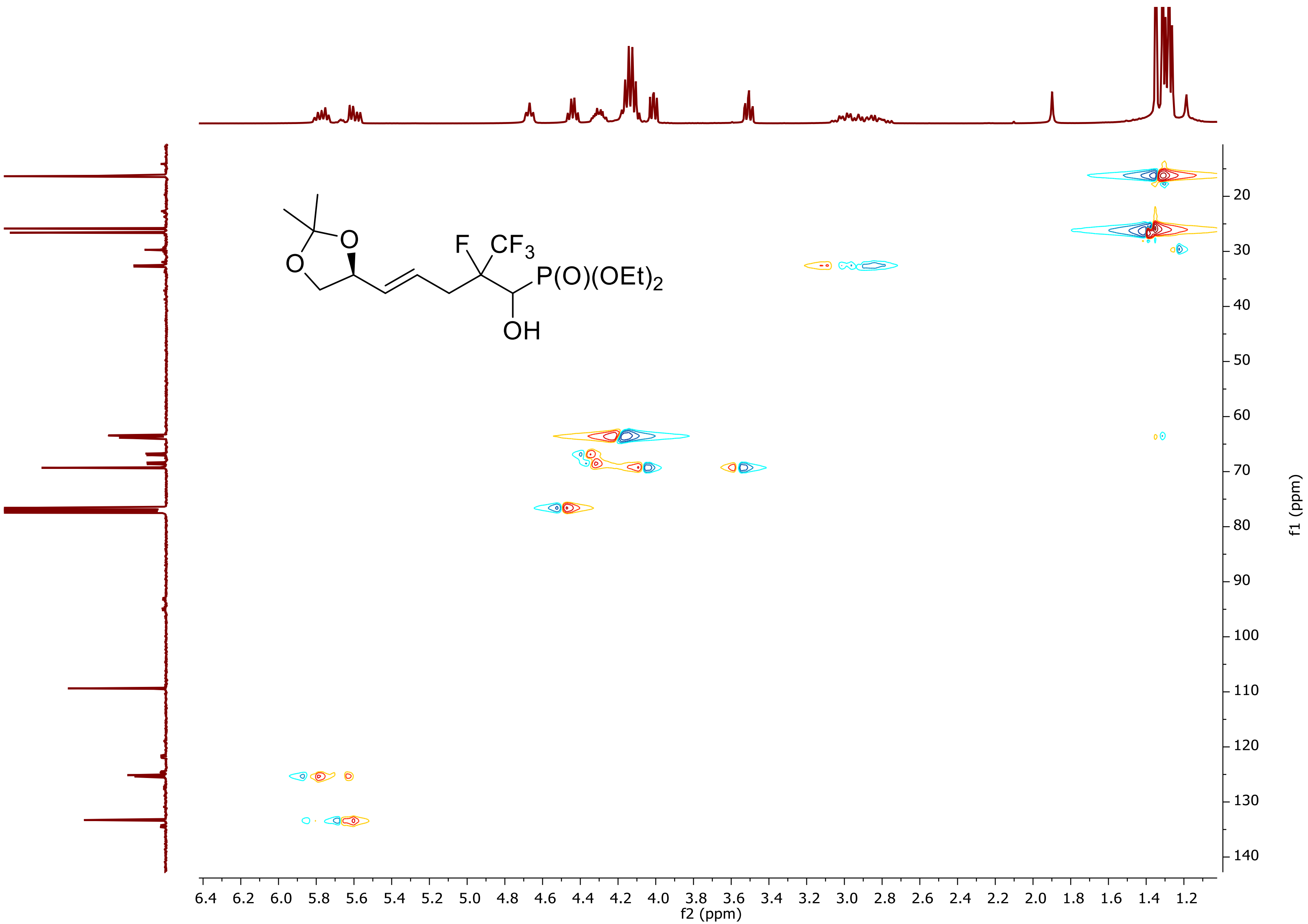

^{1}H-^{13}C HSQC of **21a/21a′**.

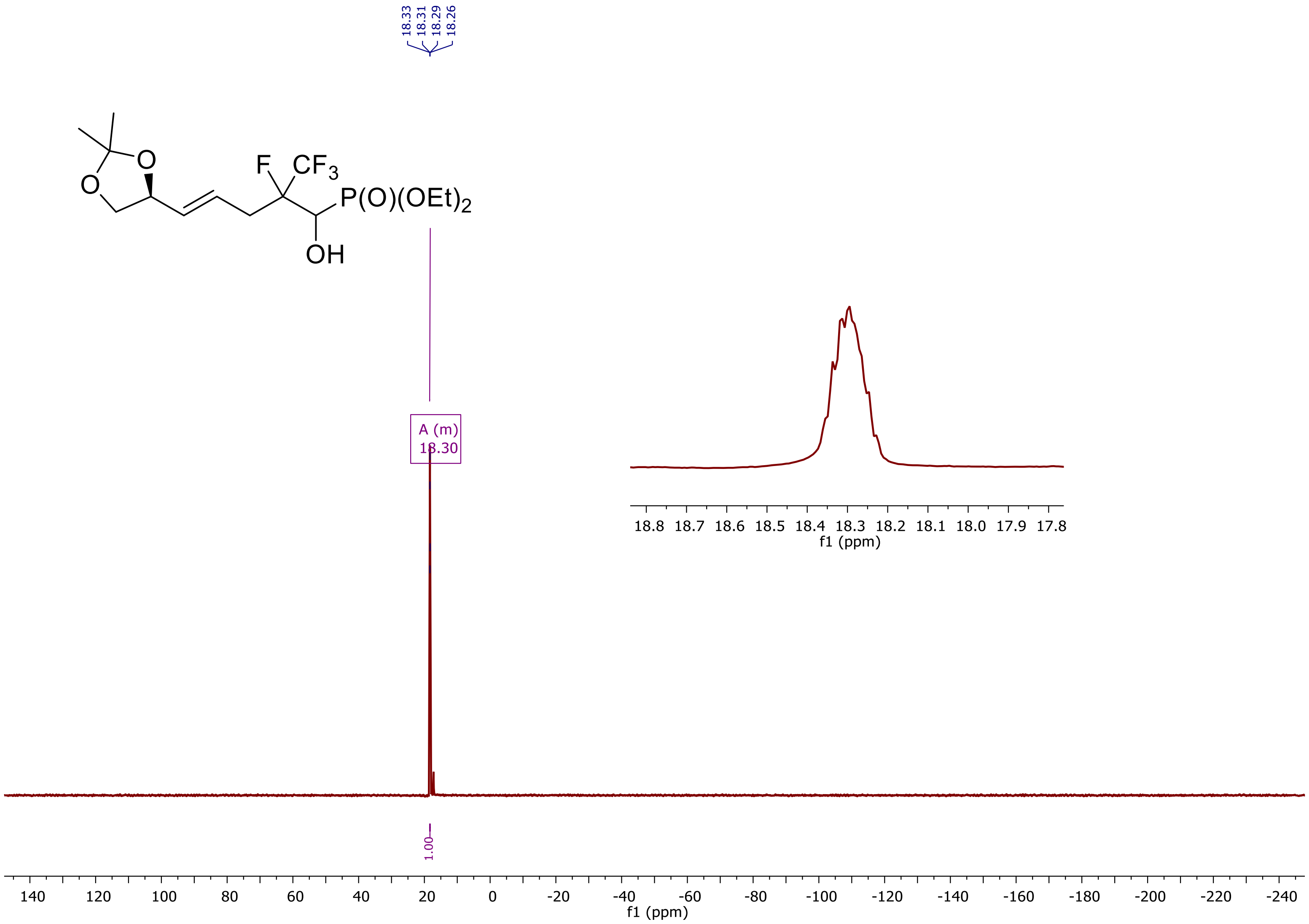

^{31}P{/^{1}H} NMR of **21a/21a′**.

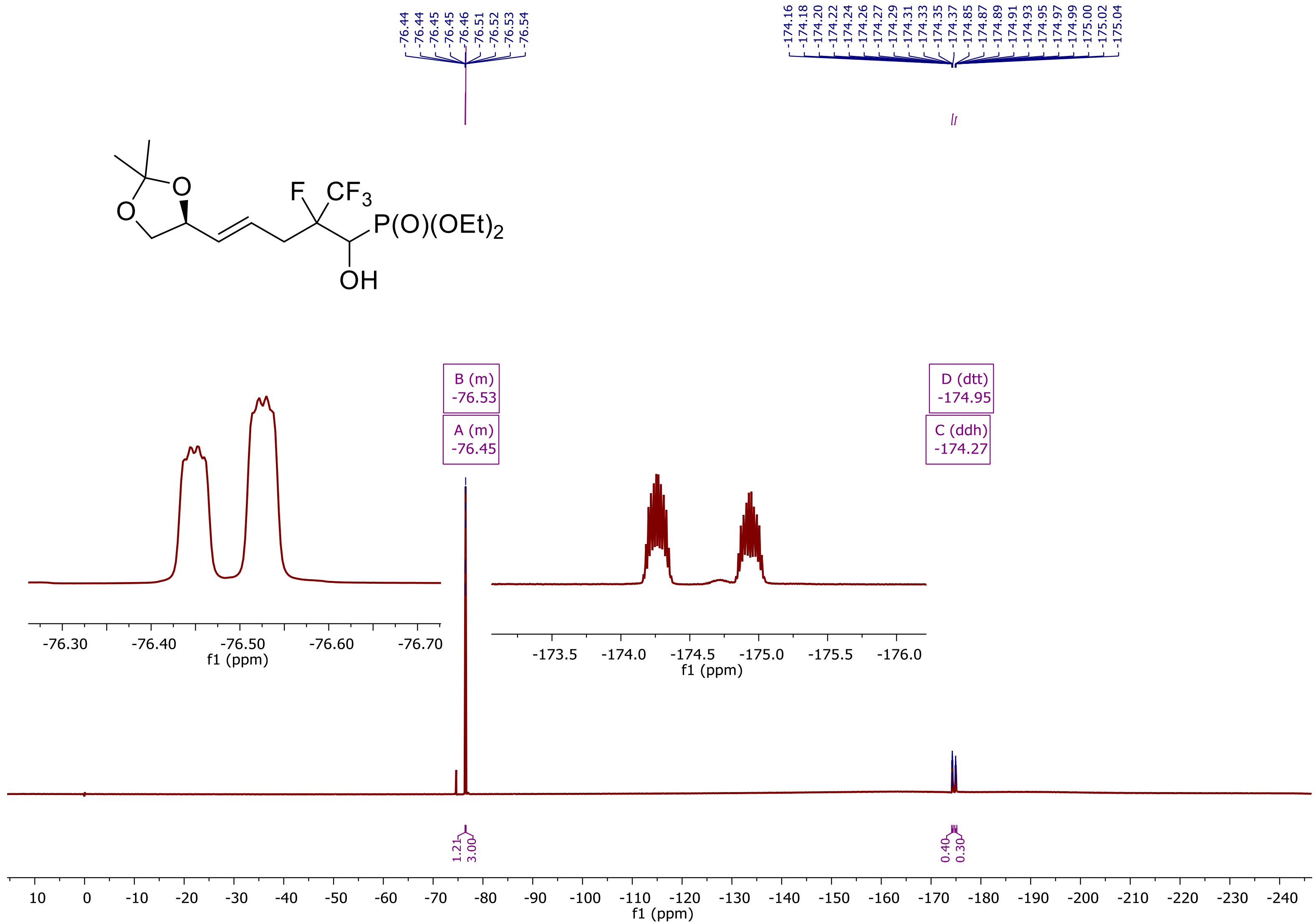

^{19}F NMR of **21a/21a′**.

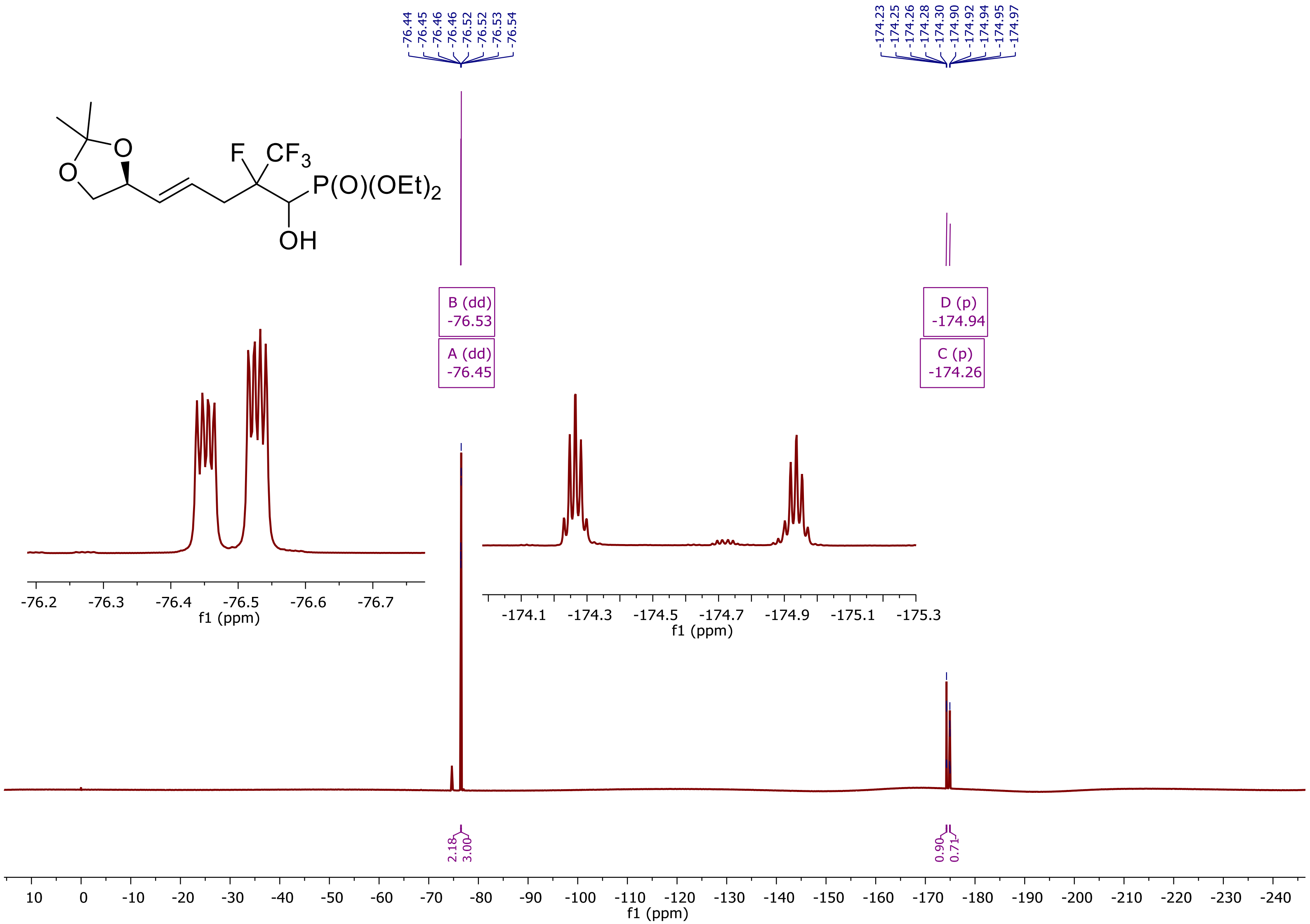

$^{19}F\{^{1}/H\}$ NMR of **21a/21a′**.

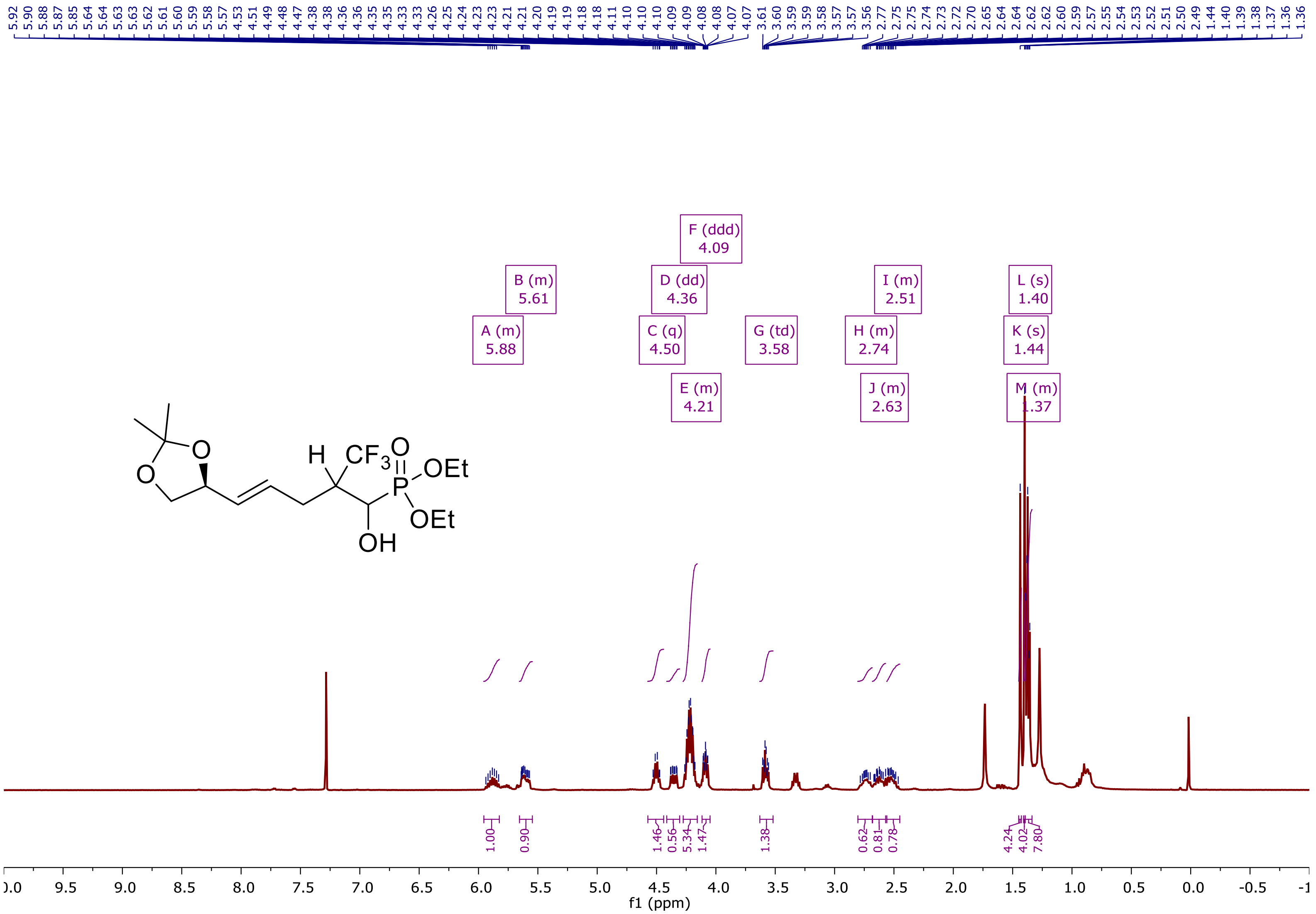

^{1}H NMR of **21b/21b′**.

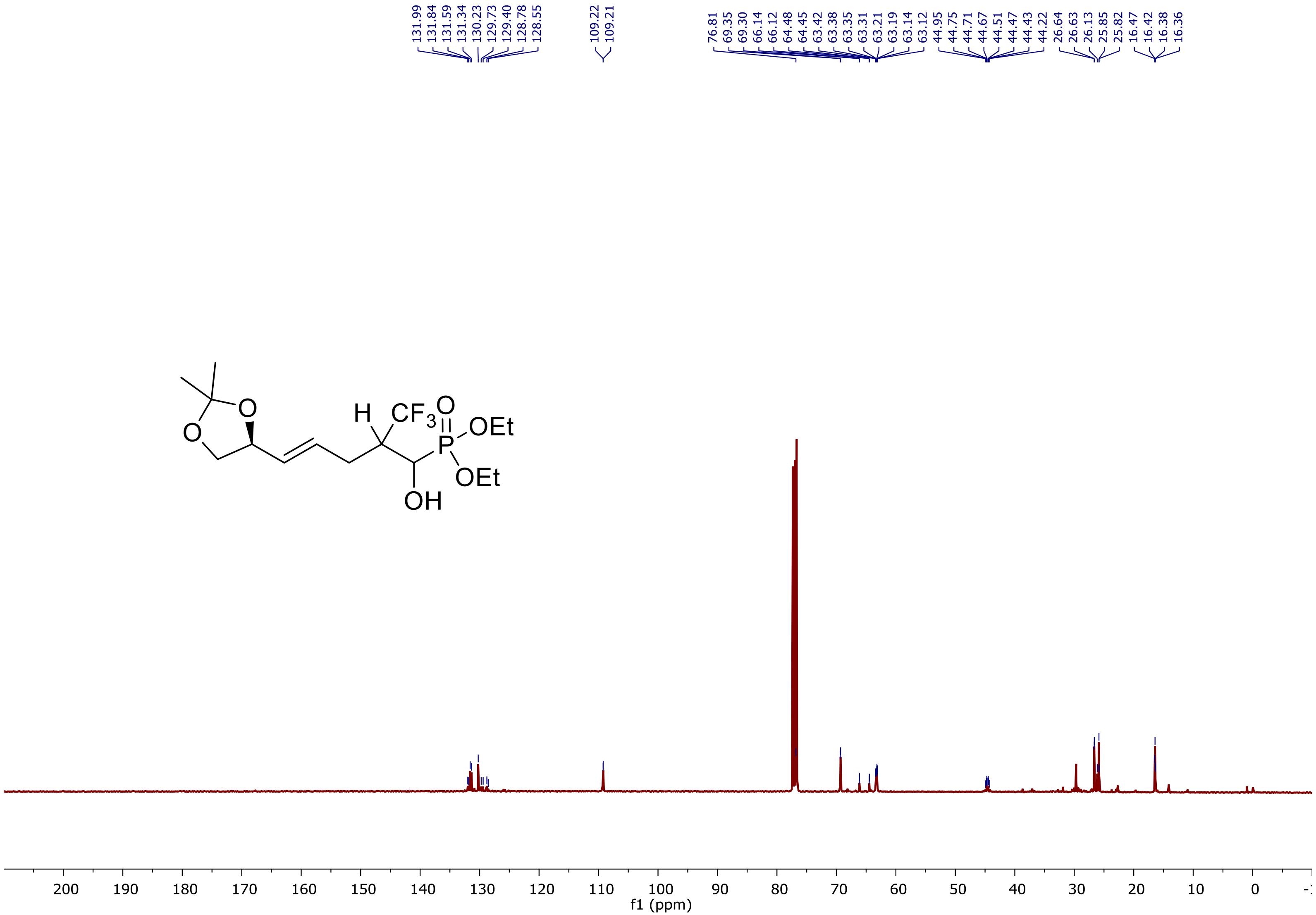

^{13}C NMR of **21b/21b′**.

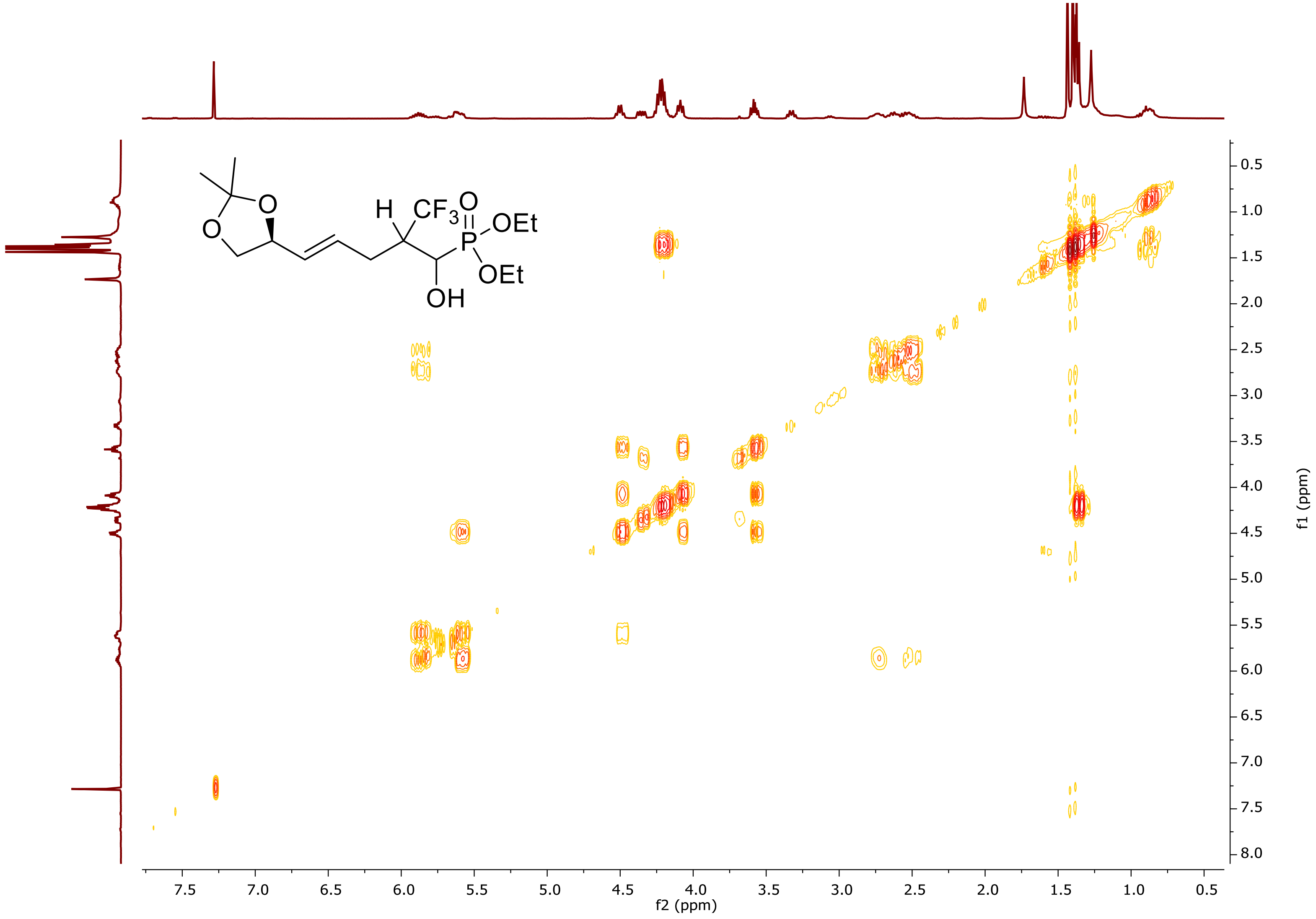

^{1}H-^{1}H COSY of **21b/21b′**.

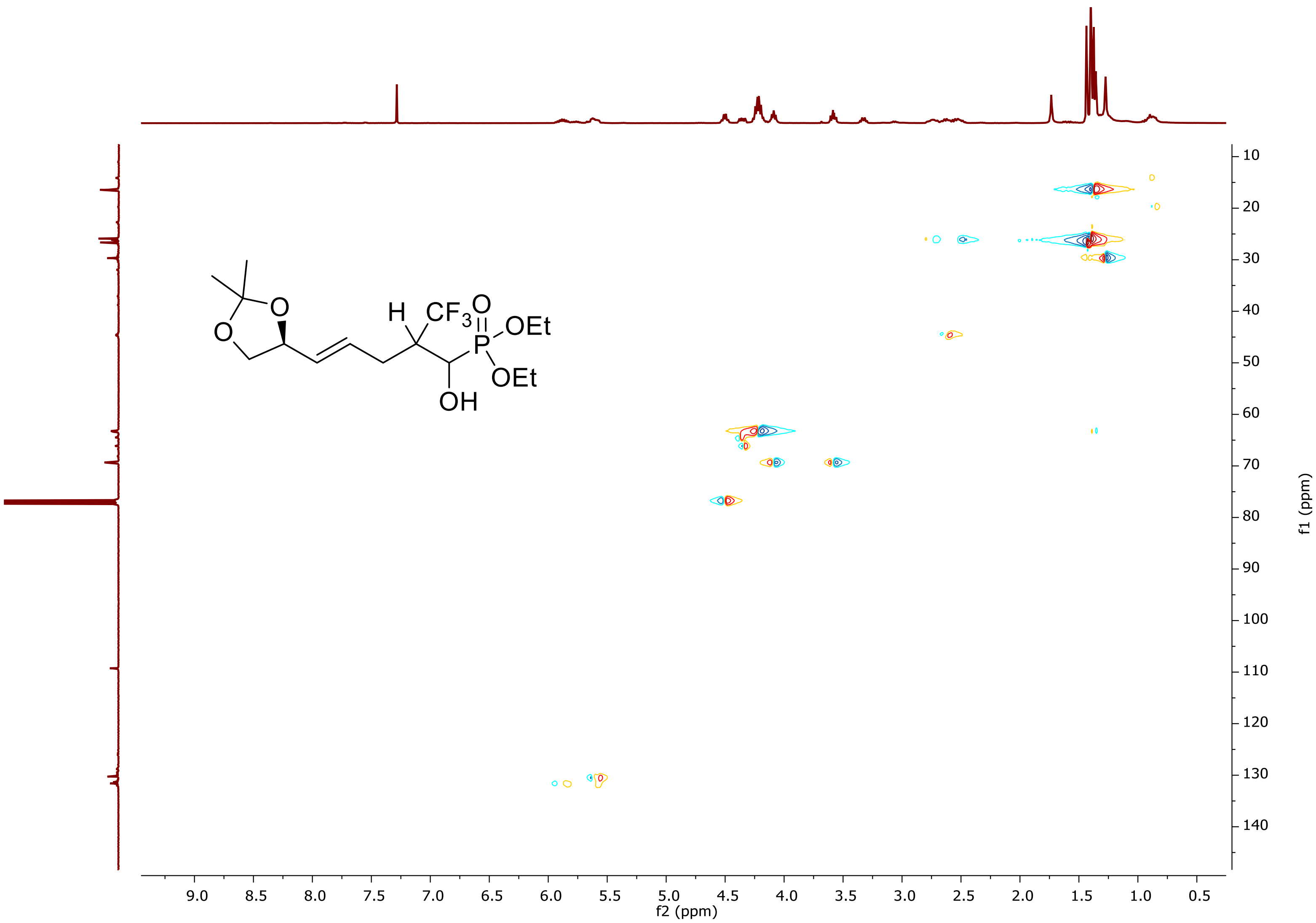

^{1}H-^{13}C HSQC of **21b/21b′**.

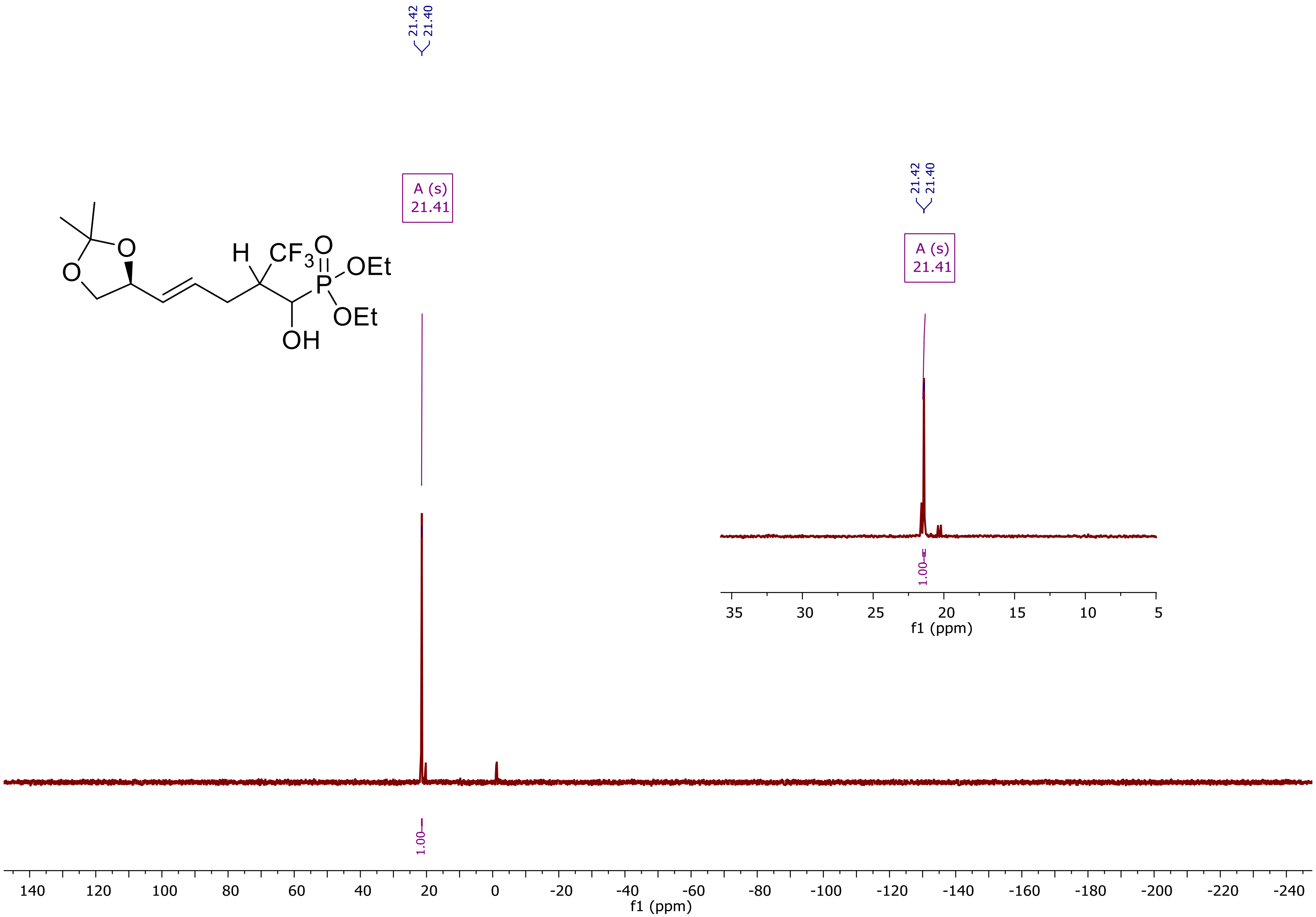

$^{31}P\{/^{1}H\}$ NMR of **21b/21b′**.

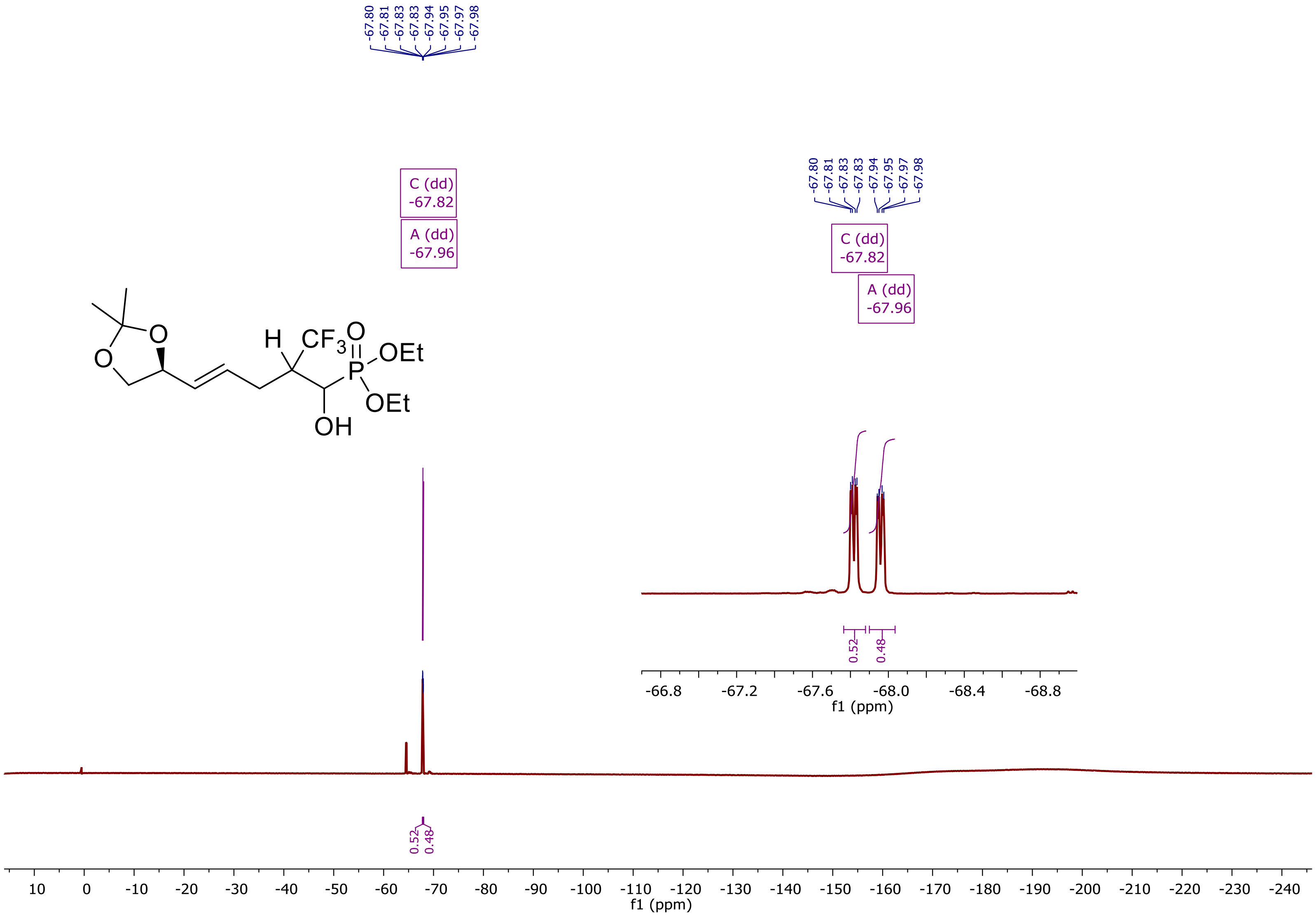

^{19}F NMR of **21b/21b′**.

DFT Coordinates:

Table S1. Atomic coordinates of optimized structures of 2-((2,3,3,3-tetrafluoroprop-1-en-1-yl)oxy)but-3-enal and α-trifluoromethyl-γ,δ-unsaturated aldehyde.

	2-((2,3,3,3-tetrafluoroprop-1-en-1-yl)oxy)but-3-enal				α-trifluoromethyl-γ,δ-unsaturated aldehyde		
Atom	x	y	z	Atom	x	y	z
C	2.33614	1.15888	-0.69054	C	-3.08070	0.11411	1.53163
O	2.93098	2.15071	-0.36601	C	-2.14662	0.37412	0.34925
H	1.86923	1.03575	-1.70098	C	-0.80956	-0.28344	0.46275
C	2.17660	-0.04699	0.22959	C	0.33350	0.38745	0.60085
C	3.30971	-0.99239	-0.00650	C	1.67126	-0.27193	0.78119
C	4.45373	-0.93909	0.67083	C	2.70250	0.21879	-0.26285
O	0.96195	-0.73136	-0.07106	C	3.06475	1.66558	0.02878
C	-0.15949	-0.06218	0.24977	H	3.87246	1.83098	0.76964
H	-0.08094	0.82218	0.88669	O	2.47516	2.59359	-0.46357
C	-1.35990	-0.47030	-0.16272	H	2.27711	0.15831	-1.27004
F	-1.52095	-1.54493	-0.93933	C	3.94604	-0.64262	-0.22815
C	-2.65199	0.19074	0.17565	F	4.89381	-0.16804	-1.05596
F	-3.48002	-0.64877	0.81321	F	3.69013	-1.90782	-0.59308
F	-3.29166	0.59903	-0.92947	F	4.48764	-0.68341	1.00519
F	-2.46061	1.25861	0.96035	O	-2.88183	-0.20491	-0.72122
H	2.17398	0.31840	1.27219	O	-4.35649	0.28200	0.94999
H	3.15527	-1.71225	-0.81579	C	-4.27470	-0.16837	-0.39948
H	4.60898	-0.21513	1.47470	C	-4.82816	-1.57791	-0.52842
H	5.28046	-1.61118	0.44043	C	-4.99263	0.83964	-1.27699
				H	-2.96800	0.83154	2.34665
				H	-2.94650	-0.90824	1.91707
				H	-2.03103	1.45706	0.19182
				H	-0.82152	-1.37472	0.45267
				H	0.31814	1.47816	0.58374
				H	2.06556	-0.06758	1.78598
				H	1.56791	-1.35877	0.69228
				H	-5.89815	-1.58286	-0.30222
				H	-4.31309	-2.24381	0.17024
				H	-4.67313	-1.95019	-1.54499
				H	-4.51916	1.81946	-1.17193
				H	-4.94242	0.52742	-2.32374
				H	-6.04111	0.91874	-0.97561

Table S2. Atomic coordinates of optimized structures of formic acid, trifluoroacetic acid and dimethyl malonate.

	Formic Acid				Trifluoroacetic Acid				Dimethyl Malonate		
Atom	x	y	z	Atom	x	y	z	Atom	x	y	z
C	-0.12288	0.37011	0.00004	C	0.96141	-0.14640	-0.00001	C	1.12268	0.54159	0.17804
H	-0.04081	1.46601	-0.00002	O	1.66955	0.96938	0.00001	O	2.43679	0.51775	-0.05167
O	1.04416	-0.28163	0.00001	H	1.11752	1.77301	0.00000	O	0.59630	1.58023	0.50748
H	1.79178	0.33976	-0.00016	O	1.44315	-1.24361	-0.00002	C	0.35248	-0.75905	0.02392
O	-1.17087	-0.22168	-0.00002	C	-0.58113	0.00689	-0.00001	H	0.50101	-1.16936	-0.97893
				F	-1.09617	-0.57797	-1.08075	H	0.71237	-1.48902	0.75441
				F	-0.95230	1.29500	-0.00042	C	-1.11461	-0.49867	0.27297
				F	-1.09607	-0.57727	1.08119	O	-1.67063	-0.68018	1.33249
								O	-1.71869	-0.03288	-0.81788
								C	-3.10727	0.29872	-0.67371
								H	-3.67517	-0.58995	-0.39269
								H	-3.22770	1.07194	0.08730
								H	-3.42321	0.66480	-1.64806
								C	3.09820	-0.68991	-0.45499
								H	4.15299	-0.43044	-0.51753
								H	2.95849	-1.47658	0.28887
								H	2.74201	-1.01674	-1.43413

Table S3. Atomic coordinates of acetone and chloroacetone.

	Acetone				Chloroacetone		
Atom	x	y	z	Atom	x	y	z
C	-0.00004	-0.61177	1.28465	C	2.27312	-0.48141	0.03970
H	0.88050	-1.26221	1.31881	H	2.34779	-1.29918	-0.68437
H	-0.88078	-1.26191	1.31900	H	2.42303	-0.91008	1.03723
H	0.00017	0.05698	2.14634	H	3.05049	0.25956	-0.15050
C	-0.00004	0.18035	0.00000	C	0.91632	0.18798	-0.01990
C	-0.00004	-0.61177	-1.28465	C	-0.25826	-0.78555	-0.06310
H	-0.88078	-1.26191	-1.31900	H	-0.17389	-1.49467	0.76551
H	0.00017	0.05698	-2.14634	H	-0.21151	-1.35446	-0.99660
H	0.88050	-1.26221	-1.31881	O	0.77670	1.38407	-0.02509
O	0.00011	1.39917	0.00000	Cl	-1.83745	0.01177	0.02878
